# Morphological Studies of Composite Spin Crossover@SiO_2_ Nanoparticles

**DOI:** 10.3390/nano11123169

**Published:** 2021-11-23

**Authors:** Yue Zan, Lionel Salmon, Azzedine Bousseksou

**Affiliations:** Laboratoire de Chimie de Coordination, CNRS & Université de Toulouse (INPT, UPS), 205 Route de Narbonne, 31400 Toulouse, France; yue.zan@lcc-toulouse.fr

**Keywords:** reverse microemulsion, nanoparticles, core@shell, spin crossover

## Abstract

Spin crossover (SCO) iron (II) 1,2,4-triazole-based coordination compounds in the form of composite SCO@SiO_2_ nanoparticles were prepared using a reverse microemulsion technique. The thickness of the silica shell and the morphology of the as obtained core@shell nanoparticles were studied by modifying the polar phase/surfactant ratio (ω), as well as the quantity and the insertion phase (organic, aqueous and micellar phases) of the tetraethylorthosilicate (TEOS) precursor, the quantity of ammonia and the reaction temperature. The morphology of the nanoparticles was monitored by transmission electron microscopy (TEM/HRTEM) while their composition probed by combined elemental analyses, thermogravimetry and EDX analyses. We report that not only the particle size can be controlled but also the size of the silica shell, allowing for interesting perspectives in post-synthetic modification of the shell. The evolution of the spin crossover properties associated with the change in morphology was investigated by variable temperature optical and magnetic measurements.

## 1. Introduction

Since the first report in 2006 concerning the synthesis of spin crossover nanoparticles by a reverse micelle method [1], numerous other homogeneous and heterogeneous media approaches including the use of polymers, soft and hard templates, microfluidic, spray drying and flow chemistry have been also developed [2]. However, the reverse micelles technique is presently the more developed one for obtaining such nano-objects because it is easy to implement, and it is the technique of choice to obtain various sizes and shapes of nanoparticles in a controlled manner for the same compound, permitting the fine study of the relationship between size and spin crossover properties [3,4,5,6,7,8]. This method has been also used to obtain hybrid or nanocomposite materials based on spin crossover complexes [2]. In particular, the use of silica precursors such as tetraethylorthosilicate (TEOS) in addition to the coordination chemistry reagents allowed us to obtain SCO@SiO_2_ core@shell nanoparticles, which served as a platform for the formation of various types of nanocomposite materials [9]. Indeed, according to this strategy, fluorescent agents as well as metallic nanoparticles have been grafted onto the silica shell to obtain a useful SCO@SiO_2_@fluorophore [9,10] and SCO@SiO_2_@Au nanocomposites [11]. The attachment of the fluorophore onto the silica shell allowed us to include an optical probe for the detection of spin crossover properties of the core at the nanometric scale, while the grafted gold nanoparticles, thanks to a photo-plasmonic effect, were designed to reduce the necessary energy to switch between the two spin states. These investigations showed the important role of (1) the thickness of the silica shell and (2) the shape of the particles on the physical properties of the nanocomposite. In particular, the thin shell observed for bistable [Fe(H-trz)_2_(trz)]BF_4_@SiO_2_@pyrene nanocomposite allowed a strong coupling of the pyrene excimers with the spin-state of the ferrous ions, brought about, to a large extent, by the spin-transition-induced mechanical strain [12]. The group of Coronado has also demonstrated the interest in obtaining a thin SiO_2_ shell to implement these nano-objects in electrical devices. Indeed, a thick insulating shell seriously limits the conductivity through the SCO NPs interfering in the transport properties of devices, making the detection of the spin transition difficult since the switching occurs at very low conductivity levels [13]. On the other hand, the presence of a continuous and thicker shell could also be interesting in order to protect the spin crossover nanoparticles from the environment in the case of more sensitive compounds and would help to promote homogeneous and integral post-functionalization. Moreover, the nature of the shell and its thickness can also modify the spin crossover properties of the SCO core [14,15,16,17]. Several groups have reported the specific influence of silica matrices on SCO nanoparticles. Direct synthesis of 3–5 nm [Fe(Htrz)_2_(trz)](BF_4_) particles in mesoporous silica templates exhibited larger hysteresis loops than the bulk sample [18]. The confinement of the same compound in ordered mesoporous silica MCM-41 reported by Zhao et al. [19] has also evidenced important changes in the hysteresis width depending on the water content and solvent (MeOH vs. EtOH) used during the synthesis. This set of results shows not only that the size of the particles and matrix nature may have an influence, as they are very similar, but also that minute changes in interaction between the matrix and the particle have a considerable effect on the elastic interactions and the cooperativity, which may be related to H-bonding between the silica and particle surface. In any case, these studies show that sub-5-nm particles embedded in silica may retain a large SCO cooperative character. The growth of a thick silica shell on larger [Fe(Htrz)_2_(trz)](BF_4_) rods by Herrera et al. [20] showed also such a widening of the hysteresis loop, with cyclability. Concerning the morphology of particles, various sizes, from 5 nm to 100 µm, and shapes, from spherical to highly anisotropic, have been reported for this family of compounds, leading to interesting applications [2]. As an example, anisotropic [Fe(H-trz)_2_(trz)]BF_4_@gold nanorods with plasmonic properties have demonstrated a better efficiency towards the energy balance to switch the SCO compound [21]. Such a photothermal–plasmonic effect was recently improved by the implementation of Au nanostar core and a spin-crossover shell based on the same coordination polymer arising from the specific plasmonic properties of the Au nanostar [22]. Compared to other Au morphologies, the great advantage of the nanostar shape originates from the hot spots created at the branches of the nanostar, making them ideal nanostructures for efficiently converting light into heat using low-energy 808 nm laser light.

Regarding fabrication strategies for encapsulating a variety of nanomaterials inside silica shells, the Stöber synthesis and reverse microemulsion methods are two of the most common approaches [23]. For the Stöber method, a nanoshell of silica is formed around a ‘‘seed’’ of the material to be encapsulated via the hydrolysis and condensation of a sol–gel precursor [24]. This method has been studied extensively to entrap metal, semiconductor and metal oxide nanoparticles to create a variety of probes and imaging agents [25,26]. Because of the aqueous alcohol solution in which the Stöber reaction takes place, hydrophobic nanoparticles must undergo a ligand exchange in order to effectively participate in Stöber-type coating reactions. An alternative method is based on the microemulsion techniques, in which the reverse micelles are used to confine the seed particles and control the deposition of silica within the micelles [27,28,29,30,31]. Compared with the Stöber process, the control over particle size and dispersity is higher, and both hydrophilic and hydrophobic nanoparticles can be coated using the microemulsion method without ligand exchange. To obtain sol–gel silica, an alkoxysilane like TEOS is used as a silica precursor, which is hydrolyzed to the monomer Si(OH)_4_ in a polar solvent [32]. To obtain a rapid and complete hydrolysis, an acidic or basic catalyst can be used. In the condensation step, the monomers condense to form siloxane bonds accompanied with the release of water. Condensation can also occur between the alkoxysilane and the silanol group, releasing an alcohol. Like the hydrolysis reaction, the condensation reaction can also be catalyzed through the acid or base method. The hydrolysis and condensation reactions occur simultaneously. The speed of the two processes determines the structure of products. Thus, an ammonia solution is often used to catalyze the hydrolysis of TEOS, thus significantly influencing particle size and morphology [33,34,35].

In this paper we report on the effect of the experimental conditions of reverse micelle syntheses on the thickness of the silica shell and the morphology of the as-obtained SCO@SiO_2_ nanoparticles. The changing experimental parameters were the reaction temperature, the relative concentration of the water/Triton-X/cyclohexane ternary system (ω), the quantity and the insertion phase (organic, aqueous and micellar phases) of the tetraethylorthosilicate (TEOS) silica precursor and the quantity of ammonia as a catalyst, which also modify the pH of the reaction.

## 2. Materials and Methods

### 2.1. Synthesis

All chemicals and solvents were obtained from Sigma Aldrich (Saint-Louis, MO, USA) and used without any further purification.

Synthesis of [Fe(Htrz)_2_(trz)](BF_4_) nanoparticles (1): two microemulsions were prepared. Microemulsion 1: an aqueous solution of Fe(BF_4_)_2_·6H_2_O (211 mg in 0.5 mL H_2_O) was added drop by drop and under vigorous agitation to a mixture Triton X-100 (1.8 mL), hexanol (1.8 mL) and cyclohexane (7.5 mL). Microemulsion 2: an aqueous solution of 1,2,4-triazole (131 mg in 0.5 mL H_2_O) was added drop by drop and under vigorous agitation to a mixture Triton X-100 (1.8 mL), hexanol (1.8 mL) and cyclohexane (7.5 mL). After 15 min, the two clear microemulsions were quickly combined and stirred overnight at room temperature. The mixture was washed with ethanol and diethyl ether and nanoparticles were collected by centrifugation at 4000 rpm during 5 min.

We obtained the syntheses of [Fe(Htrz)_2_(trz)](BF_4_)@SiO_2_ nanoparticles (2–18) with a similar procedure to sample 1, while modifying the quantity (from 1 to 4 equivalents vs. iron) and the insertion phase of TEOS, the quantity of ammonia (25%), the ω ratio ([H20]/[Triton X-100]) and the reaction temperature following Table 1 and Table S1. The silica TEOS precursor was added either in the aqueous phase (A), in the organic phase (B), in the two starting microemulsions (C) or directly in the final microemulsion phase (D). Increasing quantity of ammonia (0, 0.062, 0.124 mL) was incorporated in the final microemulsion after 15 min of micellar exchange and then 15 min after the TEOS addition.

### 2.2. Size and Physical Properties

Transmission electron microscopy (TEM) was carried out using a 100 kV JEOL JEM-1011 (JEOL, Croissy Sur Seine, France). TEM samples were prepared by depositing on a carbon coated copper grid (400 mesh) a few drops of the nanoparticles suspended in ethanol. High-resolution scanning transmission electron microscopy (HRSTEM) images and Energy-Dispersive X-ray Spectroscopy (EDS) analyses were recorded by TEM-JEM-ARM200F Cold FEG or TEM-JEM-2100F, both equipped with an EDX spectrometer. Elemental analysis was carried out using a Flash EA1112 (Thermo Finnigan 2003, Waltham, MA, USA) apparatus. Magnetic susceptibility of nanoparticle powder samples was measured using a Quantum Design MPMS2 magnetometer (Quantum Design France, les Ulis, France) in the temperature range from 300 to 400 K under a magnetic field of 1 T with a heating/cooling rate of 2 K/min. Optical reflectivity measurements were performed on a stereomicroscope (Motic) equipped with a Moticam 1000 CCD camera (Motic, Wetzlar, Germany). The sample temperature was controlled using a Linkam THMS600 liquid nitrogen cryostat (Linkam Scientific Instruments, London, UK). The temperature change rate was 2 K/min. Thermogravimetric analysis (TGA) data were acquired using a Perkin–Elmer Diamond thermal analyzer (PerkinElmer, Waltham, MA, USA).

## 3. Results and Discussion

**Scheme 1:**
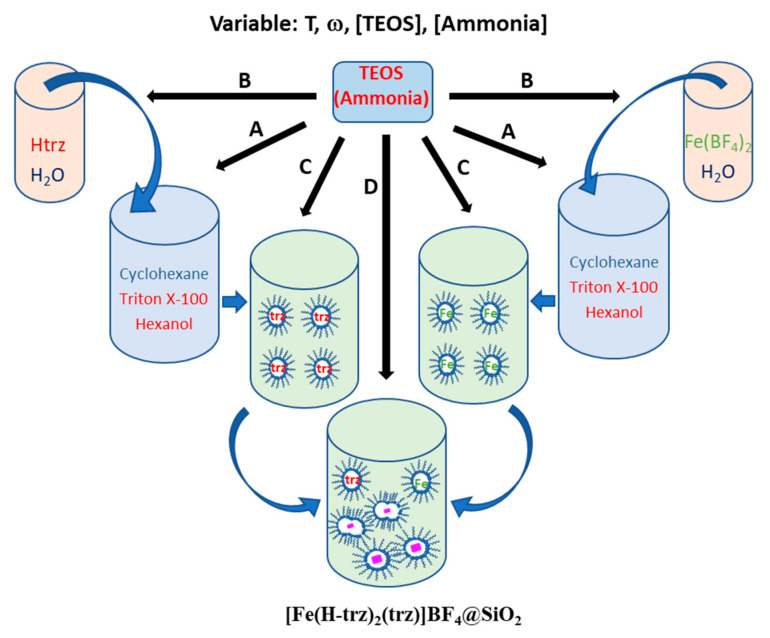
Schematic representation of the synthetic routes A–D and changing experimental parameters.

The synthetic method of the nanoparticles was inspired by S. Titos-Padilla and co-workers [9] and consisted of using reverse microemulsion with Triton X-100 as a surfactant and a co-surfactant (1-hexanol). According to Scheme 1, the TEOS silica precursor was added either in the organic phase (cyclohexane) containing hexanol as co-surfactant (synthetic route A), in the aqueous phase with the coordination complex reagents (synthetic route B), in the starting microemulsions (synthetic route C) or directly in the final microemulsion following micellar exchange (synthetic route D).

In a first series of experiments, synthetic route A was carried out using an increasing quantity of TEOS (0–0.4 mL) while maintaining all the other parameters fixed (with ω corresponding to the [water]/[surfactant] ratio, the temperature and the reaction time). Table 1 gathers all the experimental conditions for the different nanoparticle syntheses.

Particle sizes were determined by transmission electron microscopy (TEM), while their properties were probed by optical and magnetic measurements. Tentative composition of the samples was obtained thanks to combined C, H, N, Fe, and B elemental analysis and thermogravimetric analysis. Indeed, although elemental analyses confirm the formation of the SCO compounds and the presence of the silica shell, more accurate composition cannot be determined because of possible surfactant and solvent residuals.

**Figure 1:**
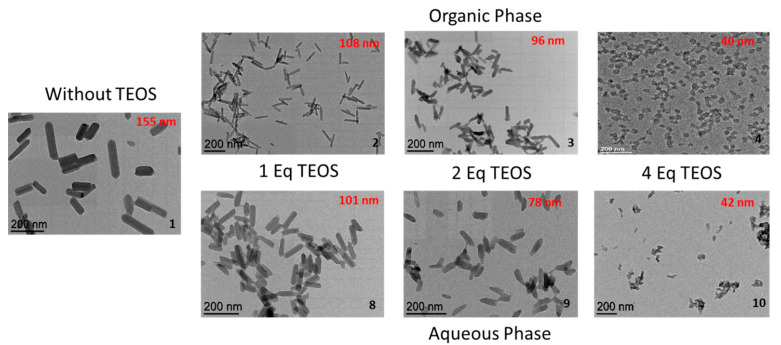
TEM images of the [Fe(Htrz)_2_(trz)](BF_4_)@SiO_2_ nanoparticles obtained without (reference sample) and with various quantity of TEOS. TEOS is inserted either in the organic phase (synthetic route A, 2–4) or in the aqueous phase (synthetic route B, 8–10). The mean value of the length of nanoparticles is given.

The TEM image of the reference [Fe(Htrz)_2_(trz)](BF_4_)·0.2H_2_O sample 1 synthesized without TEOS shows nanoparticles with platelet-like morphology with average length of ~155 nm and width of ~43 nm (Figure 1). This anisotropic morphology agrees with that obtained for similar syntheses [9,10]. Figure 1 also shows a TEM image of the nanoparticles obtained in incorporating the TEOS in the organic phase with increasing quantity of TEOS from 1 to 4 equivalent compared to the amount of the iron salt. Interestingly, a clear decrease in the size of nanoparticles was observed while the quantity of TEOS increased (from L = 155 nm without TEOS to L = 40 nm for 4 eq of TEOS). This tendency, which was not fully expected, could be related to the localization of the TEOS molecules at the interface of reverse micelles, which could act directly on the size of the reverse micelles; the increase in the quantity of TEOS favored smaller-sized micelles and thus smaller nanoparticles. Indeed, as already reported, the TEOS molecules would hydrolyze at the interface of the reverse micelles, and more highly hydrolyzed TEOS species become more hydrophilic. Therefore, as the hydrolysis progresses, the partially hydrolyzed species become surface active and associated with the reverse micelle all the time [36].

Thermogravimetric analysis revealed different water amounts ranging from 0.1 to 1.2 versus iron atom (Figure S1). Using this quantity of water and thanks to the various elemental analyses (Fe, B, C, H, N), composition of the samples and in particular the quantity of SiO_2_ around the particles could be estimated, as shown in Table 2 and Table S2. 

Increasing the quantity of TEOS slightly increased the amount of SiO_2_ in the final compound, as evidenced in particular when passing from 1 eq (sample 2) to 2 eq (sample 3) of TEOS. Such a tendency was also confirmed by the Energy Dispersive X-Ray (EDX) analyses coupled to the transmission Electronic Microscopy, which showed an increase in the %Si from ca. 26% (2) to ca. 38% (3) versus %Fe measured for a large set of nanoparticles (Figure S2). Bright-field HRSTEM images and the corresponding EDX distribution of Fe and Si along the cross-section line for nanoparticle sample 3 clearly reveals the Fe@Si structure with a silica shell, which can be estimated at 3 nm in line with reported data (Figure S2) [10]. This result is also in agreement with previous results concerning various core@silica nanoparticles (with core = Au or Fe_3_O_4_ nanoparticles) for which the increase in the quantity of TEOS leads to an increase in the silica shell [30,31]. Thermogravimetric analyses also allow us to compare the thermal stability of the SCO@silica nanoparticles. It has been shown that for the thicker silica shell, the inner particle becomes more stable, accounting for a smaller weight loss [30]. The similar weight losses around 70% obtained for this series of samples tend to show that the increase in the shell thickness remains rather limited (Figure S1). This result corroborates the quantity of SiO_2_ estimated for sample 4 (4 eq of TEOS) by elemental analyses and EDX analyses (30%Si vs. %Fe), which does not significantly increase in comparison with sample 3. All samples were also characterized by infrared spectroscopy (Figure S3). Clearly, all samples exhibit similar spectra to that corresponding to the [Fe(Htrz)_2_(trz)](BF_4_) bulk sample, particularly with vibrational modes at 1536 and 1497 cm^−1^, which can be attributed to the stretching deformation of the protonated and unprotonated triazole ligands, respectively [37]. On the other hand, the vibration modes of SiO_2_ are hidden by those of the complex.

The same series of experiment with increasing concentration of TEOS was realized while introducing the TEOS silica source in the aqueous phase in order to check if such a strategy permits us to increase the thickness of the shell of silica around the spin-crossover nanoparticles. As for synthetic route A, synthetic route B emphasizes the decrease in the size of nanoparticles with the increase in the quantity of TEOS (Figure 1). On the other hand, the comparison of the composition of samples synthesized following synthetic routes A and B does not allow us to conclude a significant increase in the thickness of the silica shell when TEOS is added in the aqueous phase, as suggested by the similar quantity of SiO_2_, which varies with the size of the particles. This result agrees well with the assumption that the TEOS molecules are located near the surfactant at the interface of reverse micelles. Similarly to the synthetic route A, a limited increase of the silica shell thickness was obtained while the quantity of TEOS increased.

**Figure 2:**
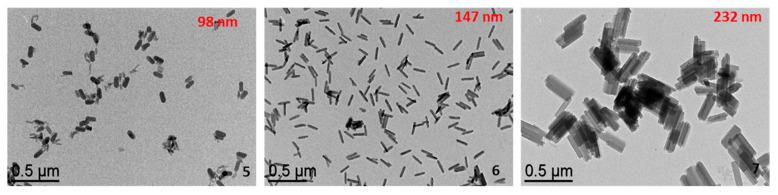
TEM images of the [Fe(Htrz)_2_(trz)](BF_4_)@SiO_2_ nanoparticles obtained at various temperature = 273 K (5), 303 K (6) and 333 K (7) for 1 eq of TEOS added in the organic phase (synthetic route A).

Figure 2 shows the effect of the temperature on the size of nanoparticles synthesized with 1 eq of TEOS introduced in the organic phase (synthetic route A). The increase in temperature from 273 to 333 K led to a significant increase in the size of nanoparticles from L = 98 nm to L = 232 nm. The effect of temperature in the growth of nanoparticles during synthesis depends on several factors, such as synthesis protocols and the nature of precursors, stabilizers and solvents. These parameters play a key role in the nucleation and growth processes associated with the formation of the nanoparticles and can be manifested by opposite effects (increase or decrease in size while heating). In our case, the increase in temperature seems to favor the growth of the particles, whose shape is not affected.

**Figure 3:**
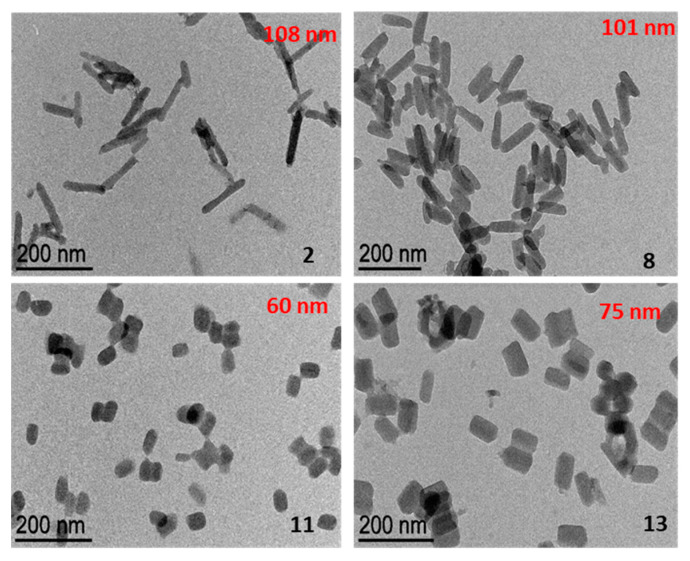
TEM images of the [Fe(Htrz)_2_(trz)](BF_4_)@SiO_2_ nanoparticles obtained following the different synthetic routes A (2), B (8), C (11) and D (13) (for ω = 10 and with 1 eq of TEOS).

The next study consisted in probing in more detail the influence of the TEOS insertion phase on the morphology of the obtained nanoparticles. We have already seen that the insertion of the TEOS in the polar or apolar phases has no significant influence on the size and SiO_2_ shell thickness of the spin crossover nanoparticles. In the same experimental conditions as samples 2 and 8 (same T°, ω, quantity of TEOS), TEOS was introduced also either in the independent starting microemulsion (synthetic route C) or directly in the final microemulsion following the micellar exchange (synthetic route D). In Figure 3, we can observe a significant decrease in the size of the nanoparticles in latter cases with nanoparticle lengths of 60 nm for the synthetic route C and 75 nm for the synthetic route D. Such a size difference reinforces the suggested role of the TEOS on the size of the reverse micelles and thus on the morphology of the core@shell nanoparticles. It appears that the TEOS influences the size of the reverse micelles even after they are already formed and also the size/shape of the SCO nanoparticles in the reverse micelles.

In order to try to increase more significantly the thickness of the silica shell, ammonia was added, as catalyst for the hydrolysis of TEOS, directly in the final microemulsion and after the addition of the silica precursor. In fact, ammonia was added after the micellar exchange and the addition of TEOS in order to avoid the destruction/oxidation of the spin crossover complex. Indeed, tentative experiments while introducing the ammonia in the starting microemulsion or in the final microemulsion but before the TEOS addition, resulted in the destabilization of the SCO core. The thin shell of silica formed after the TEOS addition (during 15 min) seems to be sufficient to protect the spin crossover core from the ammonia. It is also interesting to notice that the addition of ammonia induces an increase in the pH in the microemulsion from 4.5 to 8.

**Figure 4:**
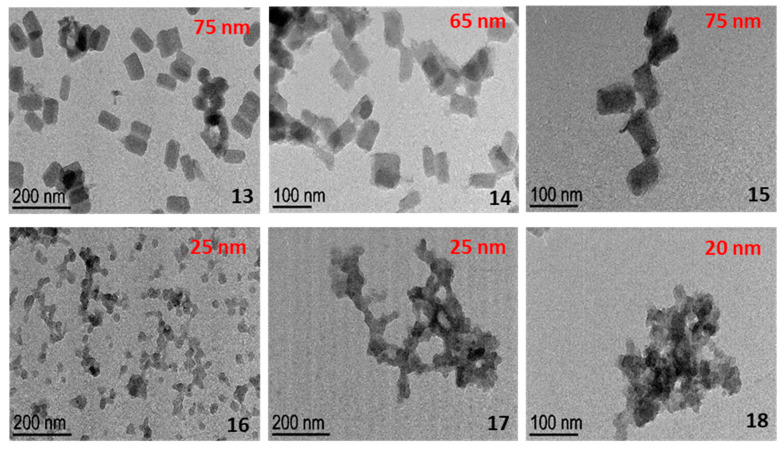
TEM images of the [Fe(Htrz)_2_(trz)](BF_4_)@SiO_2_ nanoparticles obtained following synthetic route D with ω = 10 (upper banner) and ω = 5 (bottom banner) with increasing quantity of ammonia (0, 0.062 and 0.124 mL) from the left to the right (1 eq of TEOS is used for all experiments carried out at 293 K).

Following these experimental conditions, two series of experiments were carried out modifying the ratio between surfactant and water (ω = 5 and ω = 10). As shown in Figure 4, whatever the value of ω, the increase in the quantity of ammonia up to 0.124 mL does not significantly change the size of the core shell nanoparticles. Moreover, a clear increase in the SiO_2_ amount is evidenced from the elemental and EDX analyses, which indicate for samples 15 and 18 a %Si versus %Fe of ca. 49% and 62%, respectively, for an assembly of nanoparticles; see electronic supporting information (ESI). Tentatively increasing the quantity of ammonia led to the formation of core-free silica particles. Indeed, as already reported by Ding et al., a good balance between ammonia and TEOS is essential to avoid the formation of such independent silica particles [31]. For sample 18, which contains higher amount of Si, the characteristic bands attributed to the vibration modes of SiO_2_ around 1160, 1095, 955, 800 and 470 cm^−1^, appear on the infrared spectrum and characteristic bands attributed to the SCO complex can be also observed (see ESI, Figure S3). The increase in the silica shell has an influence on the stability of the core nanoparticles, as shown by the comparison of the thermogravimetric analyses for samples 16–18. Indeed, while increasing the size of the shell, we can clearly observe a decrease in the weight losses, 70% for 16, 65% for 17 and 57% for 18.

**Figure 5:**
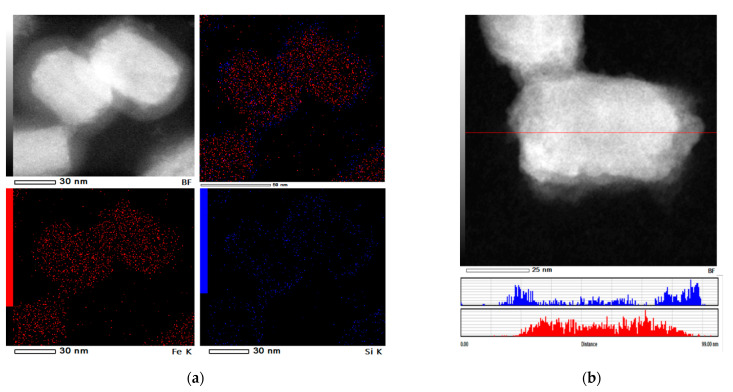
Bright-field HRSTEM images and the corresponding EDX elemental mapping images of the Fe (red), the Si (blue) and overlapped Fe and Si (**a**) and EDX distribution of Fe and Si along cross-section line (**b**) for core@shell nanoparticle sample 14.

For sample 14 (0.062 mL ammonia), the composition of the core and shell examined by EDX shows a clear distribution of Fe in the core and Si in the shell (Figure 5). The dark-field high-resolution STEM images reveal a large SiO_2_ shell of ca. 13 nm. Moreover, as expected and already reported by the group of Coronado [13], the decrease in the water-to-surfactant ratio from ω = 10 to ω = 5 is accompanied by a decrease in the size of the core nanoparticles. All attempts to further decrease the w ratio to obtain even smaller-sized nanoparticles were unfruitful because of the insolubility of the reagents.

Variable temperature optical reflectivity measurements were carried out on the different samples, and the transition temperatures for each sample are gathered in Table 2. For each sample, we consider the transition temperatures of the second thermal cycle, the first one corresponding to the dehydration of the sample. Whatever the size and the thickness of the silica shell, similar spin crossover properties are measured, in line with already reported data [6,7,8,9,10,11,12]. Indeed, for this complex, it has been shown that the size reduction has a limited effect on the spin crossover behavior since the hysteresis loop is preserved even for very small-sized (5 nm) nanoparticles [6]. Slight differences in the transition temperatures can be explained by matrix effect but also, particularly for such optical measurements on films, by the different thermal conductivity through different SiO_2_ shell thicknesses.

**Figure 6:**
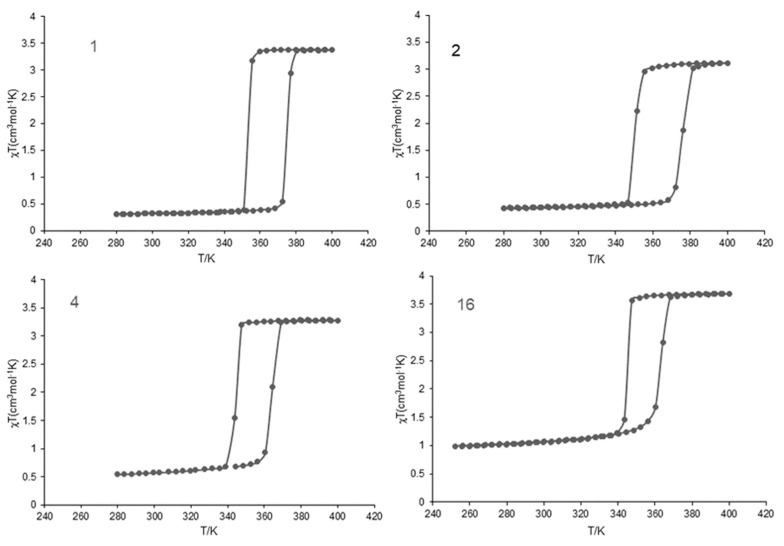
Variable temperature magnetic data for samples 1 (155 nm), 2 (108 nm), 4 (40 nm) and 16 (20 nm).

Complementary temperature-dependent magnetic susceptibility measurements were carried out on selected samples (Figure 6). The magnetic behavior shows a similar wide hysteresis of 30 K centered at ca. 360 K (T_1/2_↓ = 345 K and T_1/2_↑ = 375 K), which is very close to the corresponding bulk counterpart [37]. The χ*T* value at low temperature allows us to roughly estimate the residual high spin fraction, which increases with the decrease in the size (significant for sample 16), in agreement with the increase in the inactive surface of the nanoparticles [13].

## 4. Conclusions

In conclusion, using a reverse-micelle technique, we obtained a series of core@shell type [Fe(H-trz)_2_(trz)]BF_4_@SiO_2_ nanoparticles with lengths ranging from 250 to 20 nm while modifying well-known experimental parameters such as the temperature and the water-to-surfactant ratio. In the context of changing the nucleation and growth processes during the particle’s formation, the increase in temperature from 273 to 333 K led to a significant increase of the length of nanoparticles from ca. 100 nm to ca. 250 nm, while the decrease in ω from 10 to 5 led to a decrease of the length of nanoparticles from ca. 75 nm to ca. 20 nm. More surprisingly, we demonstrated that the quantity of TEOS and its insertion phase have a significant influence on the size of the spin crossover nanoparticles but a rather low effect on the thickness of the silica shell. In particular, a clear decrease in the length of nanoparticles was observed while increasing the quantity of TEOS from 155 nm without TEOS to 40 nm for 4 eq of TEOS. Such results tend to consider that the silica precursor localized in contact with the surfactant at the interface of the polar and apolar phases could modify the size of the micelles. On the other hand, a clear increase in the silica shell up to ca. 13 nm was obtained using ammonia as a catalyst for the hydrolysis and the condensation of TEOS. Spin-crossover properties probed by magnetic and optical measurements are similar with the previously reported bulk and nanoparticles samples. Only an increase in the residual fraction can be estimated from the magnetic measurements for the smallest (20 nm) nanoparticles. Finally, we reported that not only can the particle size be controlled but also the size of the silica shell. This possibility opens interesting perspectives according to targeted applications, since a thin shell can allow a strong interaction between the core and the molecules that can be grafted on the silica, while a thick shell can play the role of protection of the core and can also modify the mechanical properties of the composite materials.

## Figures and Tables

**Table 1:** Experimental conditions for reverse micelle syntheses of [Fe(H-trz)_2_(trz)]BF_4_@SiO_2_ nanoparticles with the Triton X-100/cyclohexane/water ternary system (volume given for the final microemulsion).

Sample	Temperature (K)	ω	TEOS Inclusion Phase (Synthetic Route)	TEOS (mL)	Ammonia (mL)
1	293	10	organic (A)	0	0
2	293	10	organic (A)	0.1	0
3	293	10	organic (A)	0.2	0
4	293	10	organic (A)	0.4	0
5	273	10	organic (A)	0.1	0
6	303	10	organic (A)	0.1	0
7	333	10	organic (A)	0.1	0
8	293	10	aqueous (B)	0.1	0
9	293	10	aqueous (B)	0.2	0
10	293	10	aqueous (B)	0.4	0
11	293	10	starting microemulsions (C)	0.1	0
12	293	5	starting microemulsions (C)	0.1	0
13	293	10	final microemulsion (D)	0.1	0
14	293	10	final microemulsion (D)	0.1	0.062
15	293	10	final microemulsion (D)	0.1	0.124
16	293	5	final microemulsion (D)	0.1	0
17	293	5	final microemulsion (D)	0.1	0.062
18	293	5	final microemulsion (D)	0.1	0.124

**Table 2:** Composition, morphological and spin crossover properties extracted from optical measurements for nanoparticle samples 1–18.

Sample	Formula	Size (nm)		T_1/2_ ↑ (K)	T_1/2_ ↓ (K)	∆T (K)
1	[Fe(Htrz)_2_(Trz)](BF_4_)·(H_2_O)_0.3_	155	43	385	350	35
2	[Fe(Htrz)_2_(Trz)](BF_4_)·(SiO_2_)_0,6_·(H_2_O)_0.5_	108	18	390	352	38
3	[Fe(Htrz)_2_(Trz)](BF_4_)·(SiO_2_)_0.9_·(H_2_O)_0.1_	96	24	396	350	46
4	[Fe(Htrz)_2_(Trz)](BF_4_)·(SiO_2_)_0.5_·(H_2_O)_0.4_	40	30	382	344	38
5	[Fe(Htrz)_2_(Trz)](BF_4_)·(SiO_2_)_0.3_·(H_2_O)_0.4_	98	42	391	346	45
6	[Fe(Htrz)_2_(Trz)](BF_4_)·(SiO_2_)_1.1_·(H_2_O)_0.4_	147	29	390	345	45
7	[Fe(Htrz)_2_(Trz)](BF_4_)·(SiO_2_)_0.6_·(H_2_O)_0.3_	232	63	392	351	41
8	[Fe(Htrz)_2_(Trz)](BF_4_)·(SiO_2_)_0.3_·(H_2_O)_0.4_	101	29	385	349	36
9	[Fe(Htrz)_2_(Trz)](BF_4_)·(SiO_2_)_0.4_·(H_2_O)_0.5_	78	26	393	349	44
10	[Fe(Htrz)_2_(Trz)](BF_4_)·(SiO_2_)_1_·(H_2_O)_0.4_	42	16	387	347	40
11	[Fe(Htrz)_2_(Trz)](BF_4_)·(SiO_2_)_0.5_·(H_2_O)_0.4_	60	35	387	354	33
12	[Fe(Htrz)_2_(Trz)](BF_4_)·(SiO_2_)_0.6_·(H_2_O)_0.6_	30	23	388	345	43
13	[Fe(Htrz)_2_(Trz)](BF_4_)·(SiO_2_)_1.1_·(H_2_O)_0.4_	75	50	378	343	35
14	[Fe(Htrz)_2_(Trz)](BF_4_)·(SiO_2_)_1.6_·(H_2_O)_0.5_	60	40	392	347	45
15	[Fe(Htrz)_2_(Trz)](BF_4_)·(SiO_2_)_2.9_·(H_2_O)_1.2_	75	37	388	334	54
16	[Fe(Htrz)_2_(Trz)](BF_4_)·(SiO_2_)_1.7_·(H_2_O)_0.5_	25	20	377	339	38
17	[Fe(Htrz)_2_(Trz)](BF_4_)·(SiO_2_)_2.7_·(H_2_O)_0.7_	25	25	382	346	36
18	[Fe(Htrz)_2_(Trz)](BF_4_)·(SiO_2_)_3.6_·(H_2_O)_1.2_	20	15	380	337	43

## Data Availability

Data can be available upon request from the authors.

## Supplementary Materials

The following are available online at https://www.mdpi.com/article/10.3390/nano11123169/s1. Table S1: Experimental conditions for the synthesis of samples 1–18. Table S2: Elemental analyses and formula for sample 1–18. Figure S1: Thermogravimetric analyses. Figure S2: Transmission Electronic Microscopy (TEM) and EDX analyses. Figure S3: Infrared spectroscopy. Figure S4: Optical reflectivity measurements for samples 1–18.
