# Supplementary material for: Morphological Studies of Composite Spin Crossover@SiO2 Nanoparticles
Source: Nanomaterials (Basel). 2021 Nov 23;11(12):3169. doi: 10.3390/nano11123169 (PMC8706634; doi:10.3390/nano11123169)
Supplement: Supplementary file 1 [file nanomaterials-11-03169-s001.zip › nanomaterials-1462544-supplementary.pdf]

*Supplementary Materials*

# Morphological Studies of Composite Spin Crossover@$SiO_2$ Nanoparticles

**Yue Zan, Lionel Salmon * and Azzedine Bousseksou ***

Laboratoire de Chimie de Coordination, CNRS & Université de Toulouse (INPT, UPS), 205 Route de Narbonne, 31400 Toulouse, France; yue.zan@lcc-toulouse.fr

* Correspondence: lionel.salmon@lcc-toulouse.fr (L.S.); azzedine.bousseksou@lcc-toulouse.fr (A.B.)

**Table S1.** Experimental conditions for the synthesis of samples 1-18.

| sample | Temperature (°C) | *$V_{H2O}$ (mL) | ω | *$V_{TEOS}$ (mL) | TEOS inclusion phase | $V_{Ammoniac}$ |
|---|---|---|---|---|---|---|
| 1 | 20 | 0.5 | 10 | 0 | --- | 0 |
| 2 | 20 | 0.5 | 10 | 0.05 | organic | 0 |
| 3 | 20 | 0.5 | 10 | 0.1 | organic | 0 |
| 4 | 20 | 0.5 | 10 | 0.2 | organic | 0 |
| 5 | 0 | 0.5 | 10 | 0.05 | organic | 0 |
| 6 | 30 | 0.5 | 10 | 0.05 | organic | 0 |
| 7 | 60 | 0.5 | 10 | 0.05 | organic | 0 |
| 8 | 20 | 0.5 | 10 | 0.05 | aqueous | 0 |
| 9 | 20 | 0.5 | 10 | 0.1 | aqueous | 0 |
| 10 | 20 | 0.5 | 10 | 0.2 | aqueous | 0 |
| 11 | 20 | 0.5 | 10 | 0.05 | starting microemulsions | 0 |
| 12 | 20 | 0.25 | 5 | 0.05 | starting microemulsions | 0 |
| 13 | 20 | 0.5 | 10 | 0.05 | final microemulsion | 0 |
| 14 | 20 | 0.5 | 10 | 0.05 | final microemulsion | 0.062 |
| 15 | 20 | 0.5 | 10 | 0.05 | final microemulsion | 0.124 |
| 16 | 20 | 0.25 | 5 | 0.05 | final microemulsion | 0 |
| 17 | 20 | 0.25 | 5 | 0.05 | final microemulsion | 0.062 |
| 18 | 20 | 0.25 | 5 | 0.05 | final microemulsion | 0.124 |

* Volume given for each starting microemulsion

**Table S2**: Elemental analyses and formula for sample 1-18

| Compound | | | C / % | H / % | N / % | B / % | Fe / % | Calculated Formula |
|---|---|---|---|---|---|---|---|---|
| 1 | | exptl | 19.98 | 1.62 | 33.37 | 2.93 | 15.29 | $[Fe(Htrz)_2(Trz)](BF_4)\cdot(H_2O)_{0.3}$ |
| | | | 20.02 | 1.49 | 33.38 | 2.81 | 15.34 | |
| | | calcd | 20.35 | 2.43 | 35.62 | 3.05 | 15.69 | |
| 2 | | exptl | 19.03 | 1.53 | 31.94 | 2.67 | 14.16 | $[Fe(Htrz)_2(Trz)](BF_4)\cdot(SiO_2)_{0.6}\cdot(H_2O)_{0.5}$ |
| | | | 18.85 | 1.43 | 31.77 | 2.69 | 14.01 | |
| | | calcd | 18.30 | 2.28 | 32.03 | 2.74 | 14.11 | |
| 3 | | exptl | 18.03 | 1.44 | 30.33 | 2.42 | 14.81 | $[Fe(Htrz)_2(Trz)](BF_4)\cdot(SiO_2)_{0.9}\cdot(H_2O)_{0.1}$ |
| | | | 18.16 | 1.60 | 30.54 | 2.42 | 14.78 | |
| | | calcd | 17.84 | 2.01 | 31.23 | 2.67 | 13.75 | |
| 4 | | exptl | 19.22 | 1.94 | 32.08 | 2.69 | 14.87 | $[Fe(Htrz)_2(Trz)](BF_4)\cdot(SiO_2)_{0.5}\cdot(H_2O)_{0.4}$ |
| | | | 19.24 | 1.91 | 32.17 | 2.59 | 14.74 | |
| | | calcd | 18.67 | 2.28 | 32.68 | 2.80 | 14.39 | |
| 5 | | exptl | 19.88 | 1.71 | 33.57 | 2.65 | 15.72 | $[Fe(Htrz)_2(Trz)](BF_4)\cdot(SiO_2)_{0.3}\cdot(H_2O)_{0.4}$ |
| | | | 19.80 | 1.50 | 33.26 | 2.57 | 15.81 | |
| | | calcd | 19.38 | 2.35 | 33.93 | 2.90 | 14.94 | |
| 6 | | exptl | 17.21 | 1.38 | 28.81 | 2.44 | 15.12 | $[Fe(Htrz)_2(Trz)](BF_4)\cdot(SiO_2)_{1.1}\cdot(H_2O)_{0.4}$ |
| | | | 17.24 | 1.40 | 28.88 | 2.44 | 14.86 | |
| | | calcd | 17.03 | 2.11 | 29.81 | 2.55 | 13.13 | |
| 7 | | exptl | 18.90 | 1.45 | 31.86 | 2.72 | 14.82 | $[Fe(Htrz)_2(Trz)](BF_4)\cdot(SiO_2)_{0.6}\cdot(H_2O)_{0.3}$ |
| | | | 18.83 | 1.48 | 31.64 | 2.65 | 14.70 | |
| | | calcd | 18.39 | 2.17 | 32.19 | 2.75 | 14.18 | |
| 8 | | exptl | 19.64 | 1.43 | 33.05 | 2.75 | 15.53 | $[Fe(Htrz)_2(Trz)](BF_4)\cdot(SiO_2)_{0.3}\cdot(H_2O)_{0.4}$ |
| | | | 19.70 | 1.56 | 33.21 | 2.65 | 15.36 | |
| | | calcd | 19.21 | 2.34 | 33.62 | 2.88 | 14.81 | |
| 9 | | exptl | 19.29 | 1.61 | 32.24 | 2.55 | 15.71 | $[Fe(Htrz)_2(Trz)](BF_4)\cdot(SiO_2)_{0.4}\cdot(H_2O)_{0.5}$ |
| | | | 19.29 | 1.56 | 32.23 | 2.47 | 15.83 | |
| | | calcd | 18.82 | 2.36 | 32.94 | 2.82 | 14.51 | |
| 10 | | exptl | 18.27 | 1.08 | 29.40 | 2.74 | 14.18 | $[Fe(Htrz)_2(Trz)](BF_4)\cdot(SiO_2)_1\cdot(H_2O)_{0.4}$ |
| | | | 18.22 | 1.14 | 29.26 | 2.72 | 13.83 | |
| | | calcd | 17.32 | 2.12 | 30.31 | 2.59 | 13.35 | |
| 11 | | exptl | 19.12 | 1.50 | 32.57 | 3.12 | 14.94 | $[Fe(Htrz)_2(Trz)](BF_4)\cdot(SiO_2)_{0.5}\cdot(H_2O)_{0.4}$ |
| | | | 19.01 | 1.44 | 32.39 | 3.20 | 15.23 | |
| | | calcd | 18.79 | 2.29 | 32.88 | 2.81 | 14.48 | |
| 12 | | exptl | 18.32 | 1.43 | 31.12 | 3.00 | 15.59 | $[Fe(Htrz)_2(Trz)](BF_4)\cdot(SiO_2)_{0.6}\cdot(H_2O)_{0.6}$ |
| | | | 18.28 | 1.46 | 31.11 | 2.93 | 15.34 | |
| | | calcd | 18.16 | 2.32 | 31.79 | 2.72 | 14.00 | |
| 13 | | exptl | 18.28 | 1.48 | 28.74 | 2.82 | 14.23 | |

| | | | | | | | | |
|---|---|---|---|---|---|---|---|---|
| | | | 18.40 | 1.75 | 28.97 | 2.83 | 14.12 | $[Fe(Htrz)_2(Trz)](BF_4)\cdot(SiO_2)_{1.1}\cdot(H_2O)_{0.4}$ |
| | | calcd | 17.08 | 2.08 | 29.89 | 2.56 | 13.16 | |
| 14 | | exptl | 16.37 | 1.38 | 27.12 | 2.30 | 12.22 | $[Fe(Htrz)_2(Trz)](BF_4)\cdot(SiO_2)_{1.6}\cdot(H_2O)_{0.5}$ |
| | | | 17.01 | 1.33 | 28.24 | 2.30 | 12.20 | |
| | | calcd | 15.98 | 1.99 | 27.98 | 2.39 | 12.32 | |
| 15 | | exptl | 17.35 | 2.41 | 19.52 | 1.97 | 12.50 | $[Fe(Htrz)_2(Trz)](BF_4)\cdot(SiO_2)_{2.7}\cdot(H_2O)_{1.2}$ |
| | | | 17.52 | 2.57 | 19.81 | 2.01 | 12.89 | |
| | | calcd | 13.24 | 1.91 | 23.16 | 1.98 | 10.20 | |
| 16 | | exptl | 17.06 | 1.58 | 26.08 | 2.62 | 13.40 | $[Fe(Htrz)_2(Trz)](BF_4)\cdot(SiO_2)_{1.7}\cdot(H_2O)_{0.5}$ |
| | | | 17.08 | 1,39 | 26.33 | 2.53 | 13.06 | |
| | | calcd | 15.68 | 1.95 | 27.45 | 2.35 | 12.09 | |
| 17 | | exptl | 15.11 | 1.38 | 22.46 | 2.23 | 12.67 | $[Fe(Htrz)_2(Trz)](BF_4)\cdot(SiO_2)_{2.7}\cdot(H_2O)_{0.7}$ |
| | | | 14.92 | 1.37 | 22.23 | 2.25 | 12.70 | |
| | | calcd | 13.76 | 1.79 | 24.09 | 2.06 | 10.61 | |
| 18 | | exptl | 13.34 | 2.01 | 19.14 | 1.62 | 13.45 | $[Fe(Htrz)_2(Trz)](BF_4)\cdot(SiO_2)_{3.6}\cdot(H_2O)_{1.2}$ |
| | | | 13.31 | 2.08 | 19.12 | 1.61 | 13.60 | |
| | | calcd | 12.28 | 1.77 | 21.50 | 1.84 | 9.47 | |

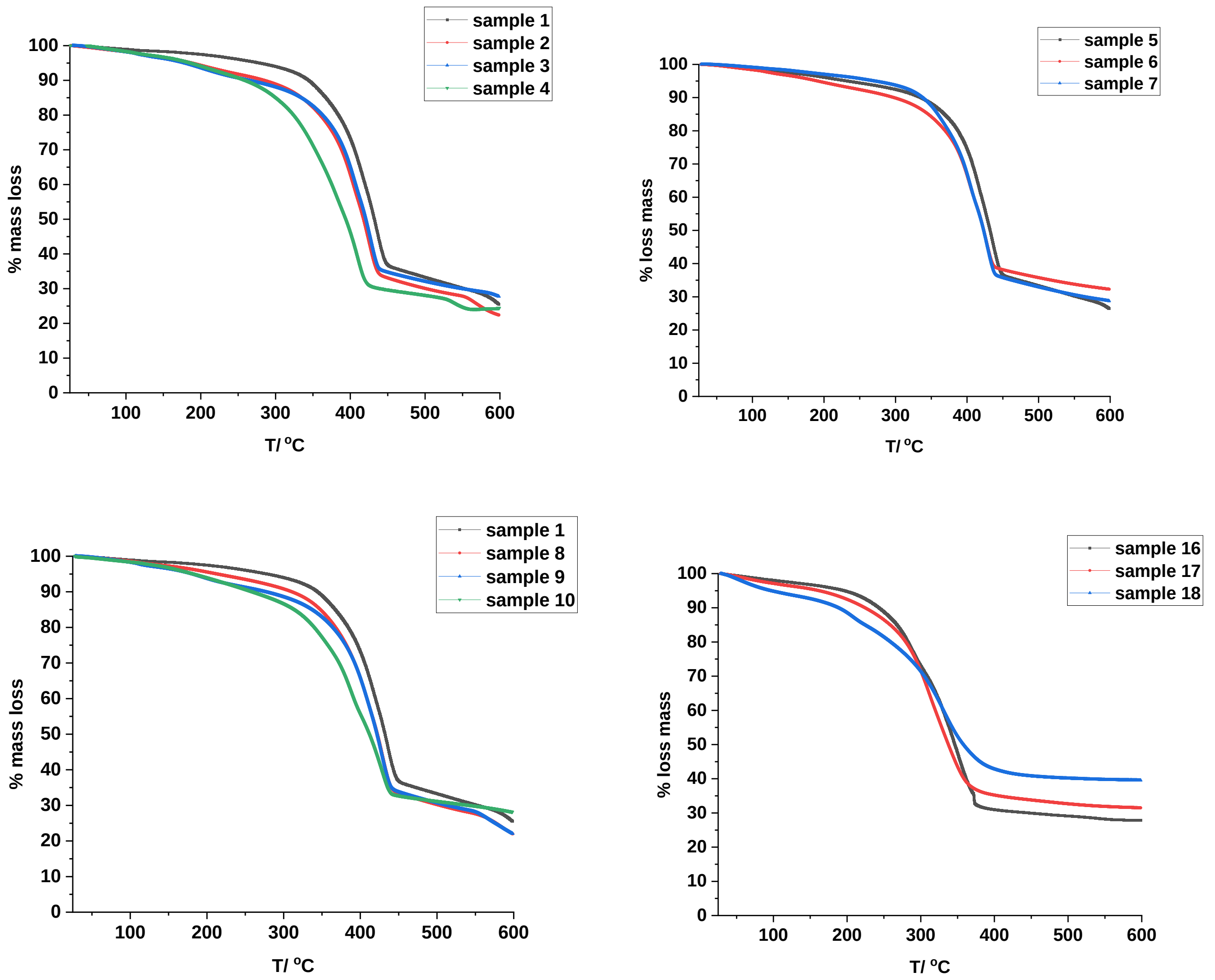


**Figure S1**: Thermogravimetric analyses

TEM images of sample 2

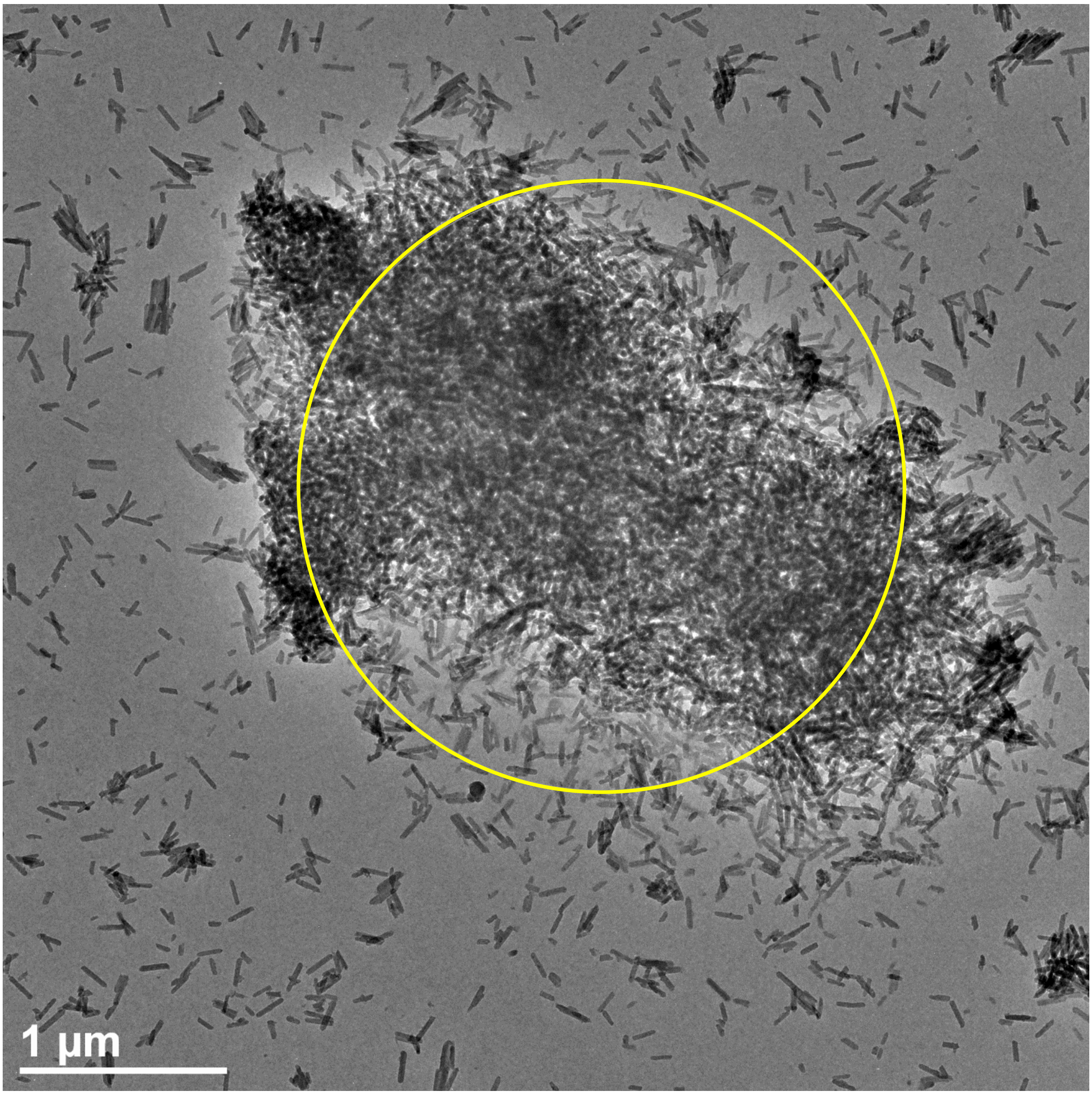


Yellow circle shows the mesured area

| Element | Series | norm. C [wt.%] | Atom. C [at.%] | Error (1 Sigma) [wt.%] |
|---|---|---|---|---|
| Silicon | K-series | 14.91 | 25.84 | 0.21 |
| Iron | K-series | 85.09 | 74.16 | 2.63 |
| | Total: | 100.00 | 100.00 | |

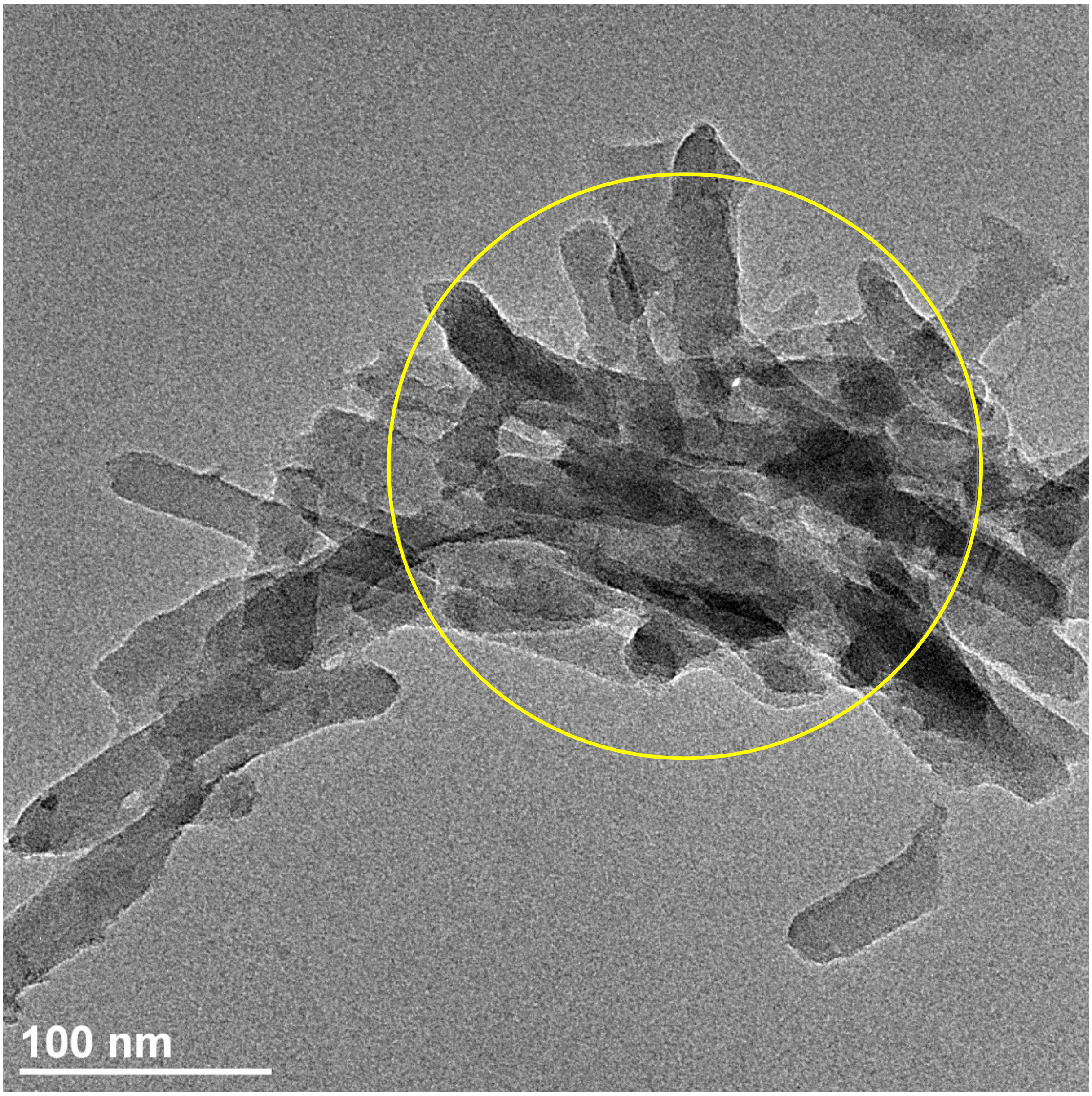


| Element | Series | norm. C [wt.%] | Atom. C [at.%] | Error (1 Sigma) [wt.%] |
|---|---|---|---|---|
| Silicon | K-series | 16.19 | 27.76 | 0.22 |
| Iron | K-series | 83.81 | 72.24 | 2.60 |
| | Total: | 100.00 | 100.00 | |

TEM images of sample 3

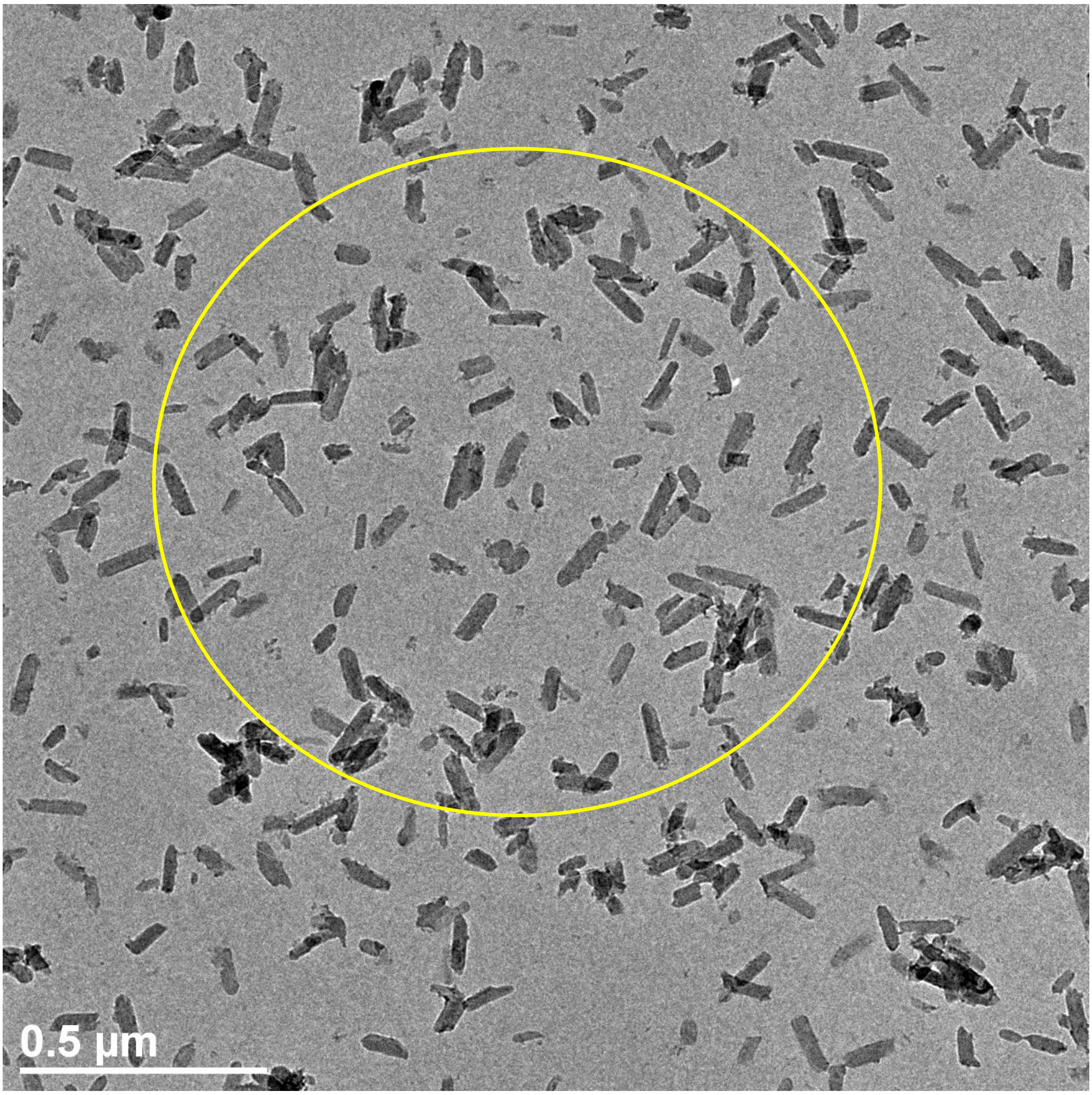


| Element | Series | norm. C [wt.%] | Atom. C [at.%] | Error (1 Sigma) [wt.%] |
|---|---|---|---|---|
| Iron | K-series | 76.25 | 61.76 | 3.37 |
| Silicon | K-series | 23.75 | 38.24 | 1.17 |
| | Total: | 100.00 | 100.00 | |

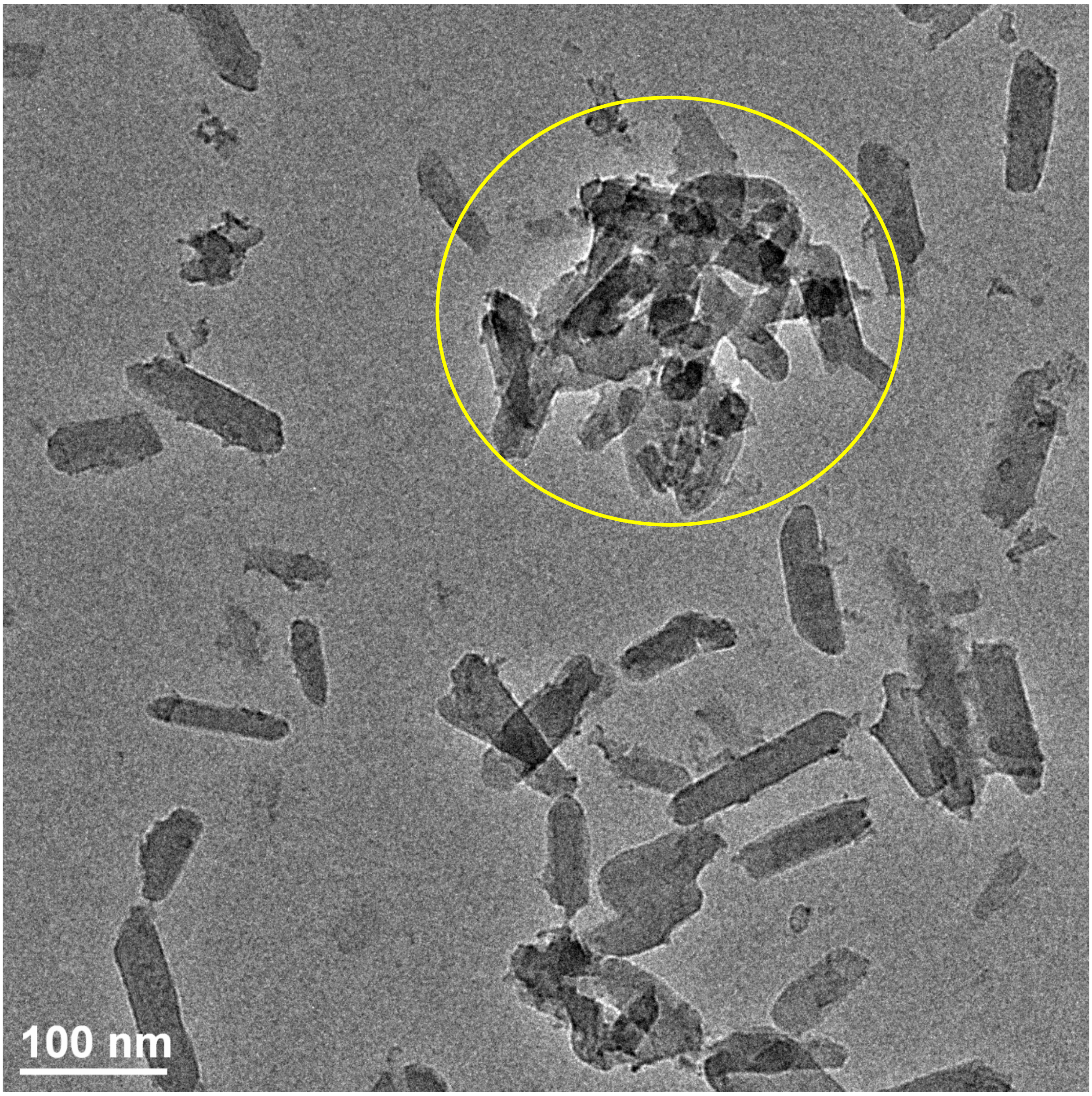


| Element | Series | norm. C [wt.%] | Atom. C [at.%] | Error (1 Sigma) [wt.%] |
|---|---|---|---|---|
| Iron | K-series | 75.42 | 60.67 | 2.68 |
| Silicon | K-series | 24.58 | 39.33 | 0.69 |
| | Total: | 100.00 | 100.00 | |

Bright field HRSTEM images and the corresponding EDX distribution of Fe (in red) and Si (in blue) along cross-section line for core@shell nanoparticle sample 3.

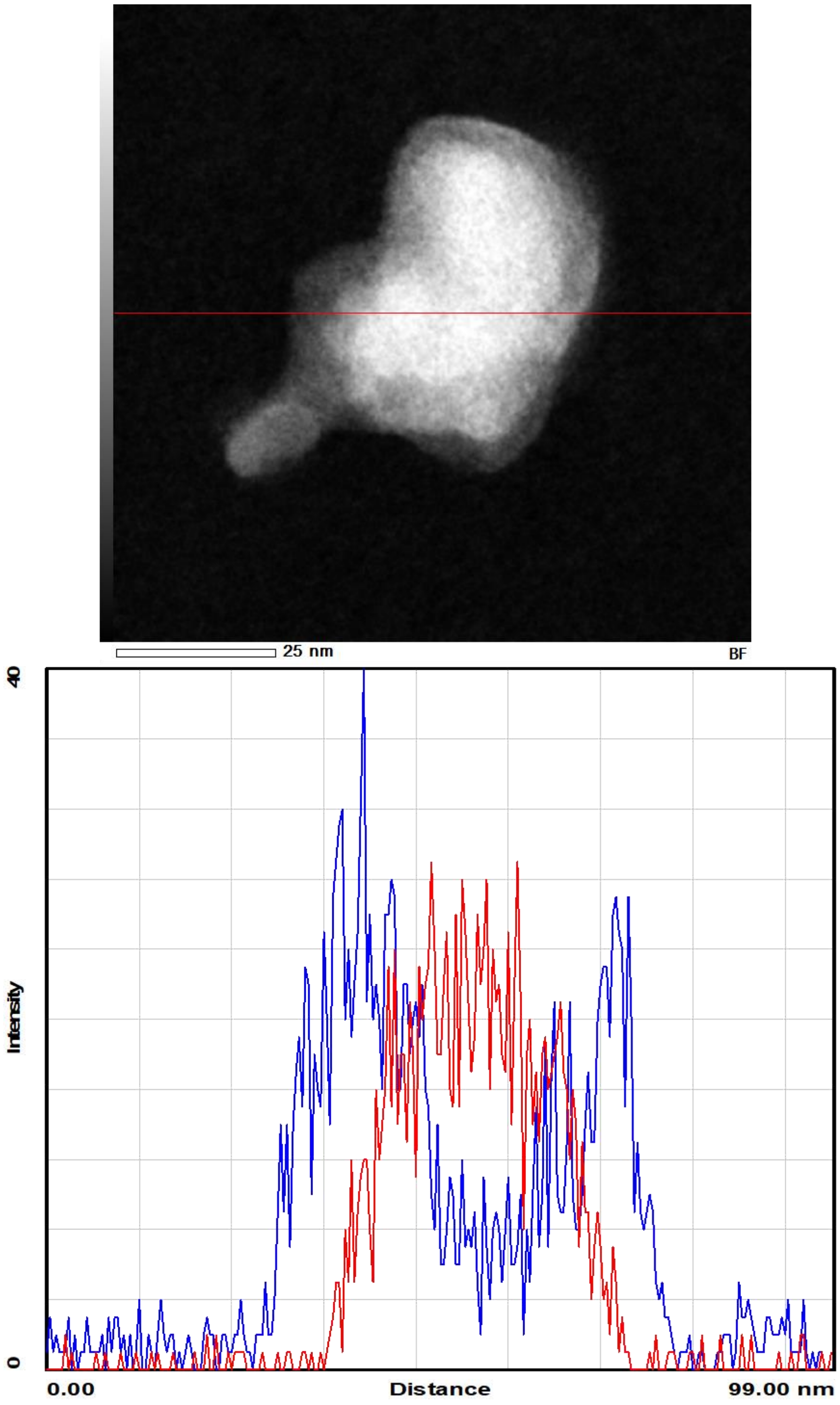

TEM images of sample 4

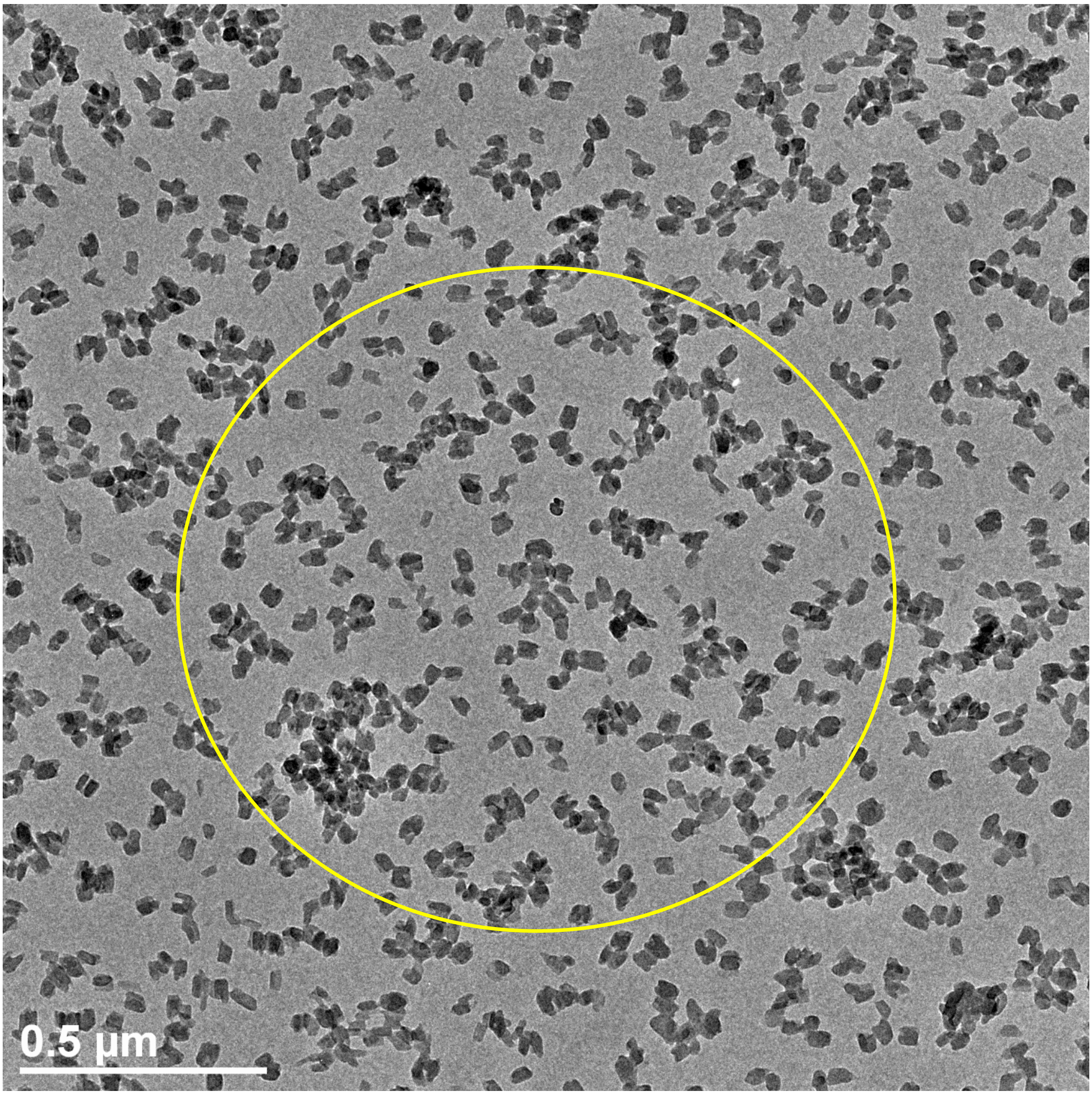


| Element | Series | norm. C [wt.%] | Atom. C [at.%] | Error (1 Sigma) [wt.%] |
|---|---|---|---|---|
| Iron | K-series | 77.75 | 63.73 | 2.60 |
| Silicon | K-series | 22.25 | 36.27 | 0.52 |
| | Total: | 100.00 | 100.00 | |

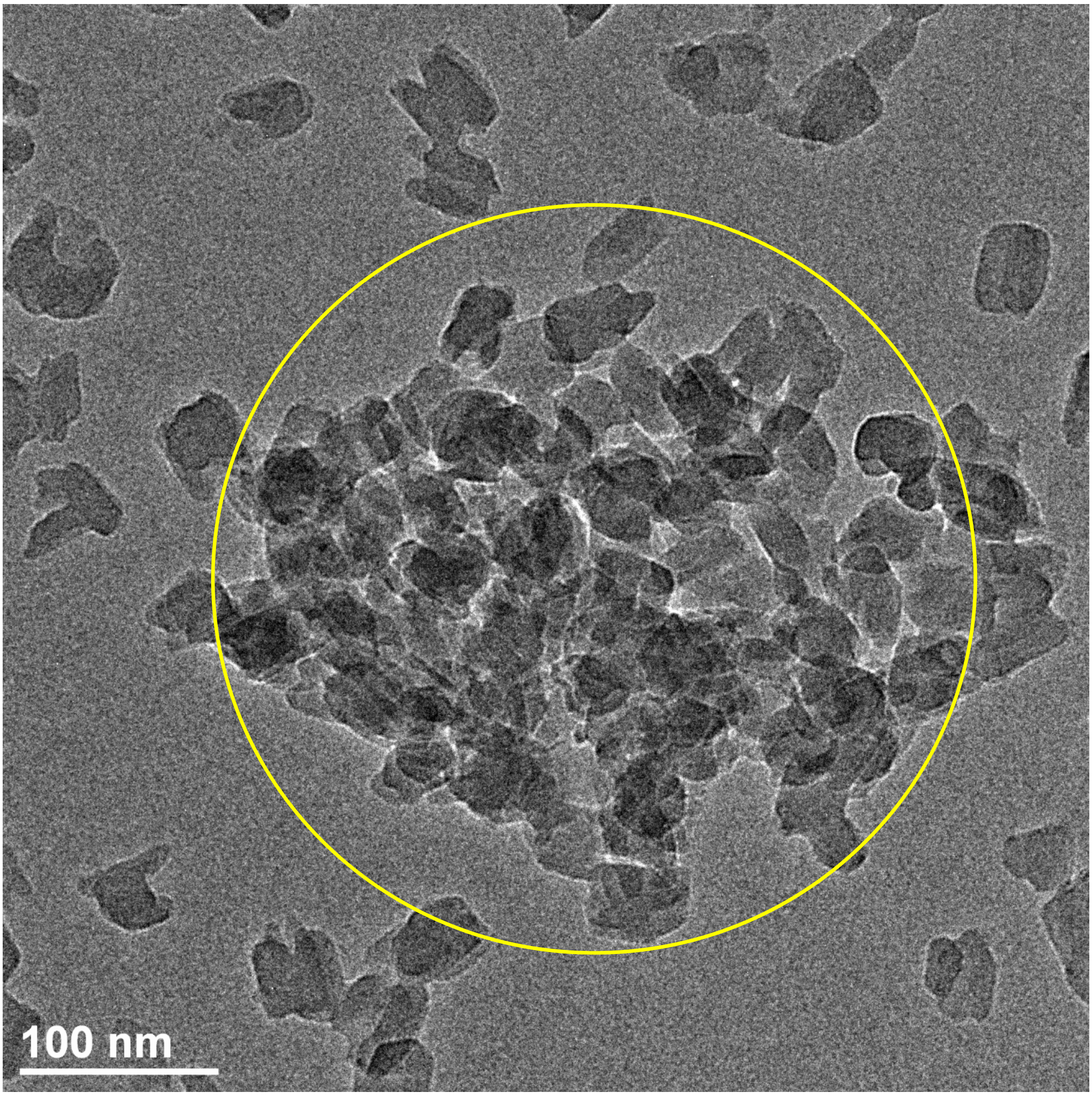


| Element | Series | norm. C [wt.%] | Atom. C [at.%] | Error (1 Sigma) [wt.%] |
|---|---|---|---|---|
| Iron | K-series | 86.60 | 76.47 | 2.65 |
| Silicon | K-series | 13.40 | 23.53 | 0.16 |
| | Total: | 100.00 | 100.00 | |

TEM images of sample 15

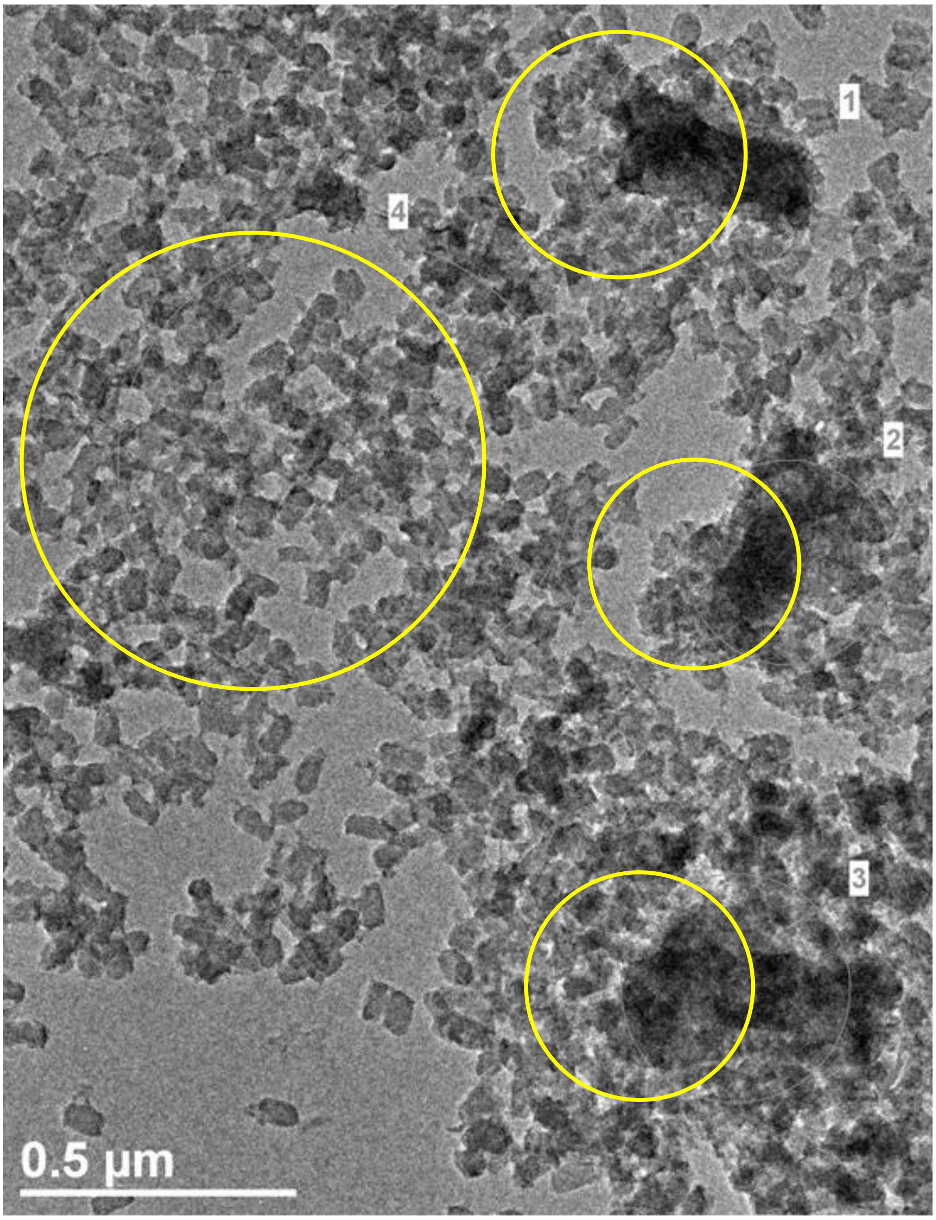


spot 3

| Element | Series | norm. C [wt.%] | Atom. C [at.%] |
|---|---|---|---|
| Silicon | K-series | 29.63 | 45.57 |
| Iron | K-series | 70.37 | 54.43 |
| | Total: | 100.00 | 100.00 |

spot 1

| Element | Series | norm. C [wt.%] | Atom. C [at.%] |
|---|---|---|---|
| Silicon | K-series | 30.53 | 46.63 |
| Iron | K-series | 69.47 | 53.37 |
| | Total: | 100.00 | 100.00 |

spot 4

| Element | Series | norm. C [wt.%] | Atom. C [at.%] |
|---|---|---|---|
| Silicon | K-series | 35.05 | 51.76 |
| Iron | K-series | 64.95 | 48.24 |
| | Total: | 100.00 | 100.00 |

spot 2

| Element | Series | norm. C [wt.%] | Atom. C [at.%] |
|---|---|---|---|
| Silicon | K-series | 31.56 | 47.83 |
| Iron | K-series | 68.44 | 52.17 |
| | Total: | 100.00 | 100.00 |

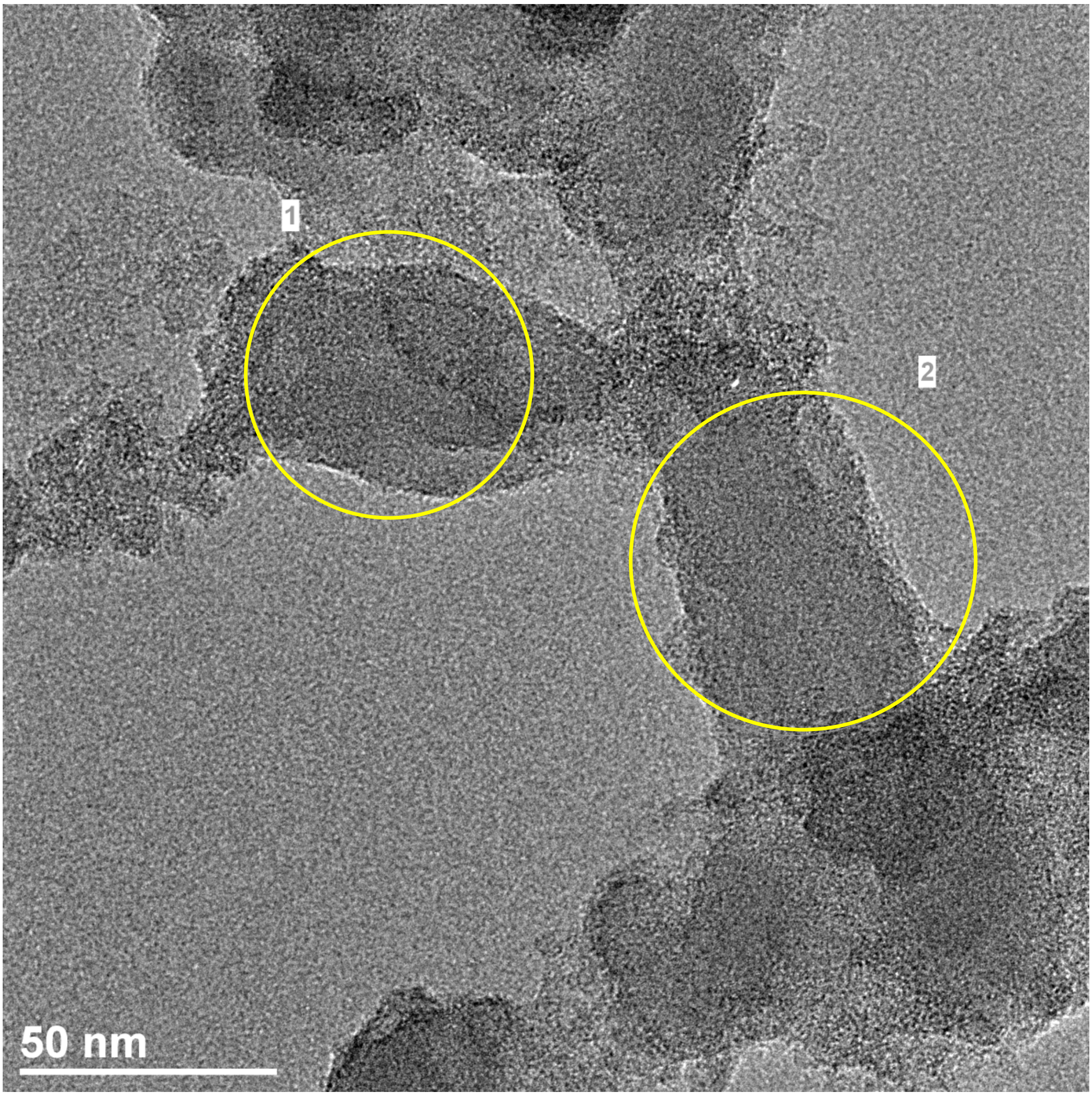


spot 1

| Element | Series | norm. C [wt.%] | Atom. C [at.%] |
|---|---|---|---|
| Silicon | K-series | 28.54 | 44.27 |
| Iron | K-series | 71.46 | 55.73 |
| | Total: | 100.00 | 100.00 |

spot 2

| Element | Series | norm. C [wt.%] | Atom. C [at.%] |
|---|---|---|---|
| Silicon | K-series | 30.58 | 46.69 |
| Iron | K-series | 69.42 | 53.31 |
| | Total: | 100.00 | 100.00 |

TEM images of sample 18

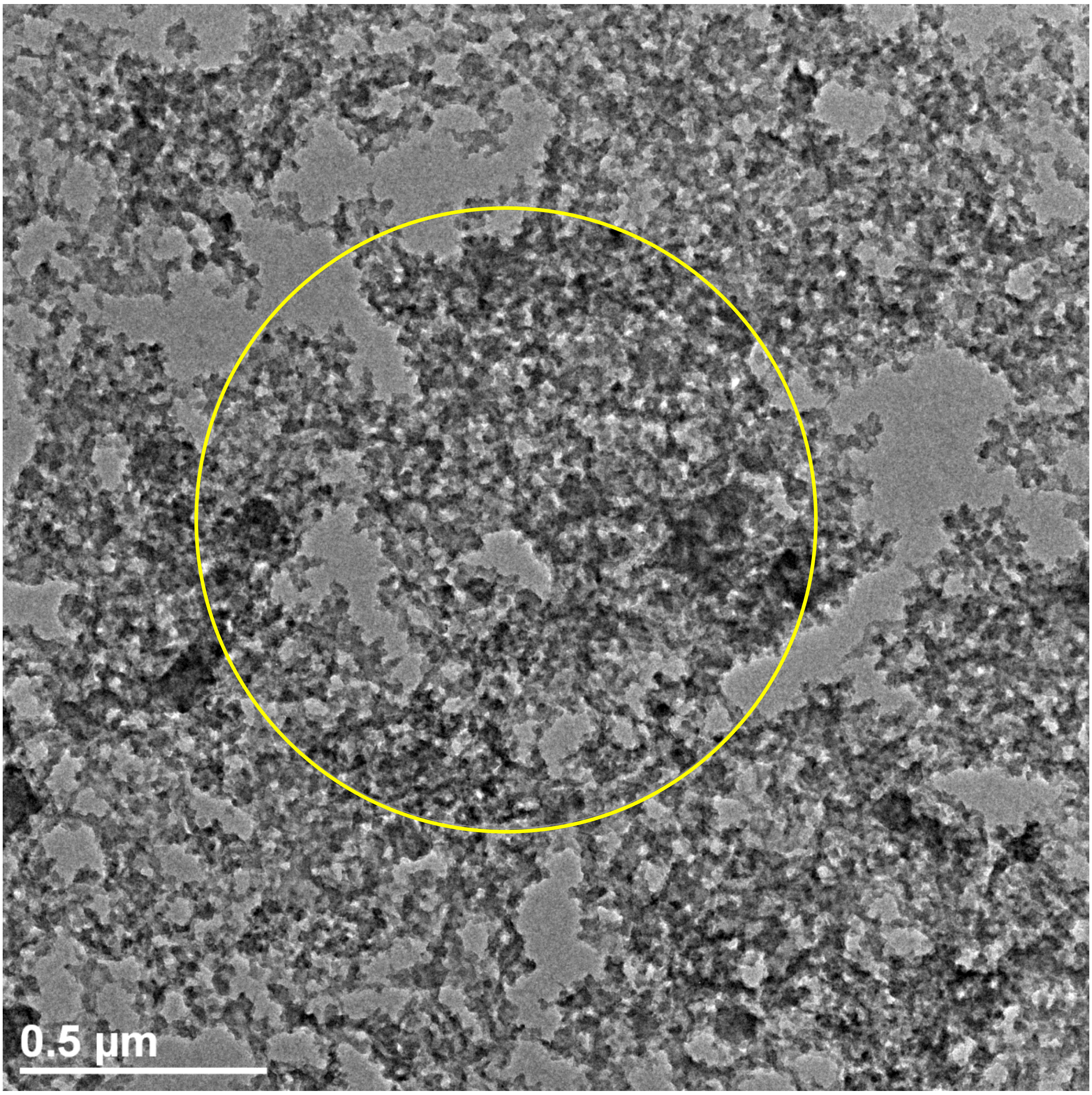


| Element | Series | norm. C [wt.%] | Atom. C [at.%] |
|---|---|---|---|
| Iron | K-series | 60.58 | 43.59 |
| Silicon | K-series | 39.42 | 56.41 |
| | Total: | 100.00 | 100.00 |

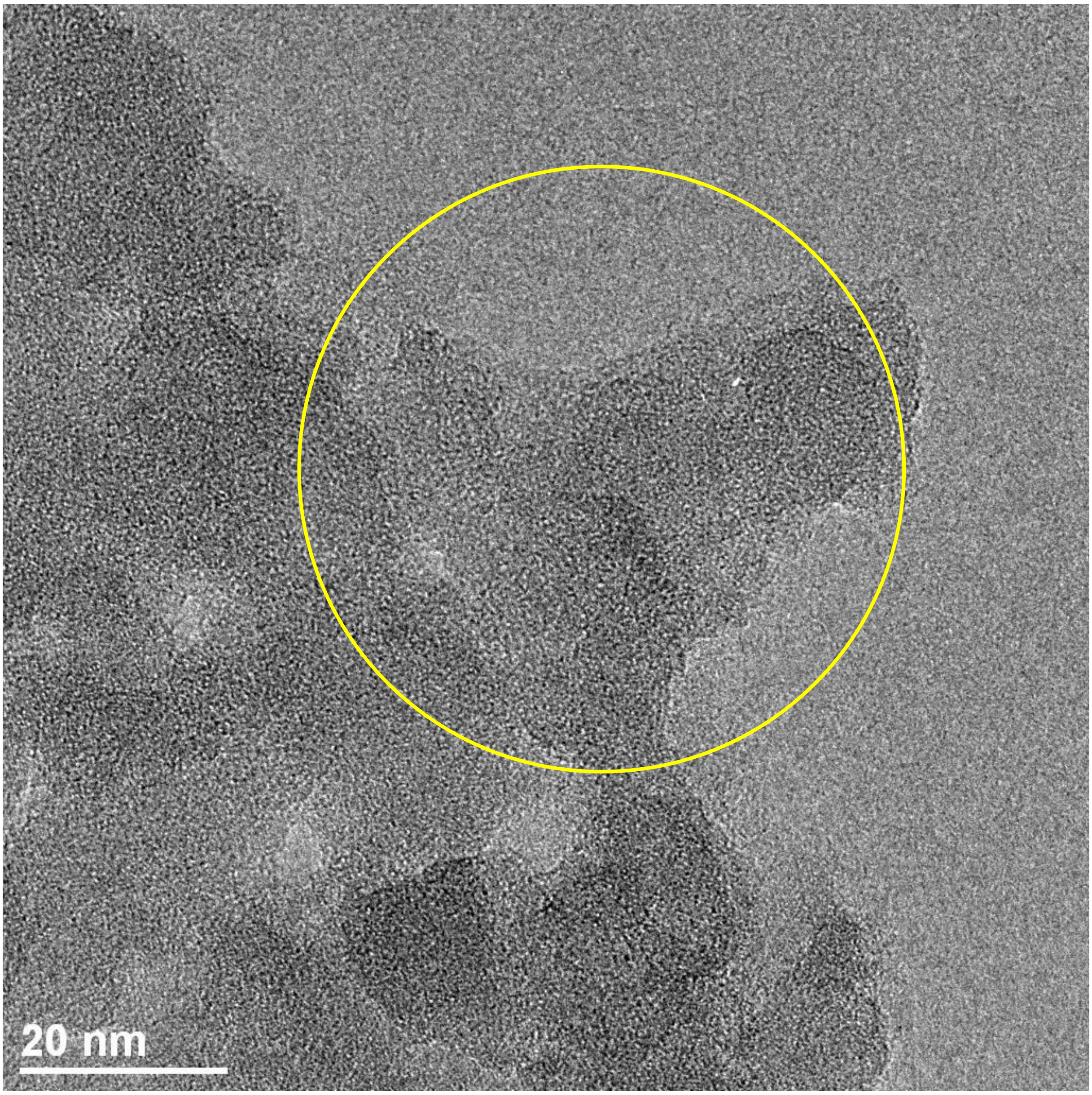


| Element | Series | norm. C [wt.%] | Atom. C [at.%] |
| --- | --- | --- | --- |
| Silicon | K-series | 48.29 | 65.00 |
| Iron | K-series | 51.71 | 35.00 |
| | Total: | 100.00 | 100.00 |

**Figure S2**: Transmission Electronic Microscopy (TEM) and EDX analyses

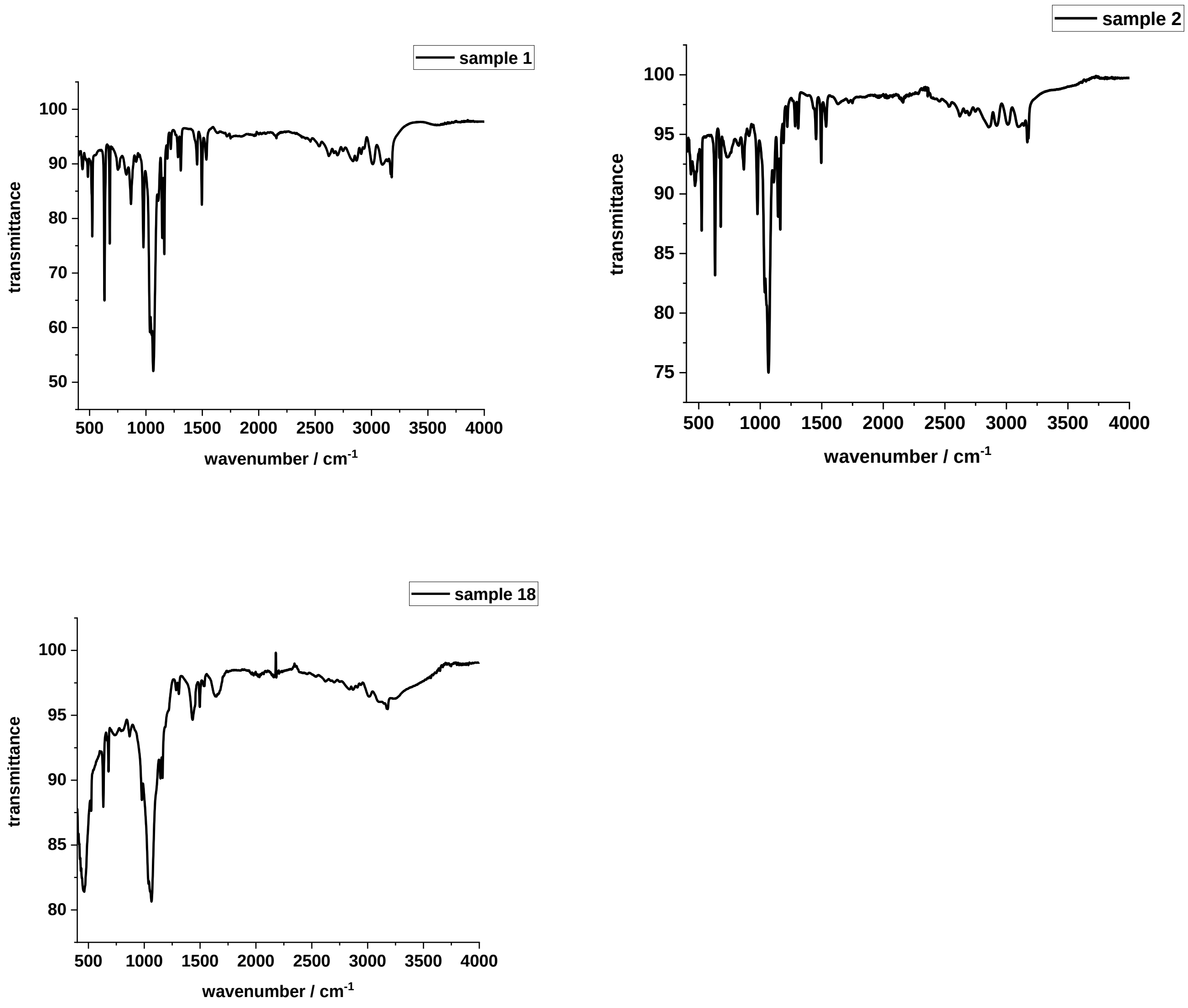


**Figure S3**: Infrared spectroscopy

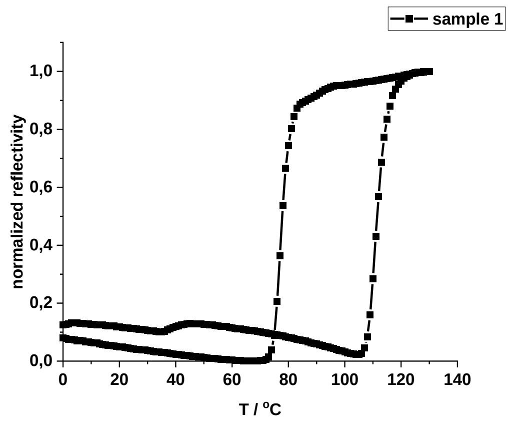
sample 1
normalized reflectivity
T / °C

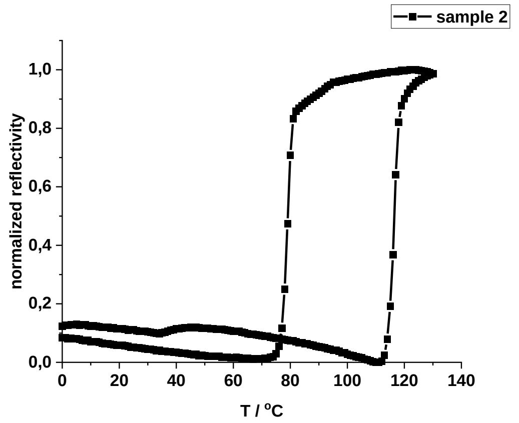
sample 2
normalized reflectivity
T / °C

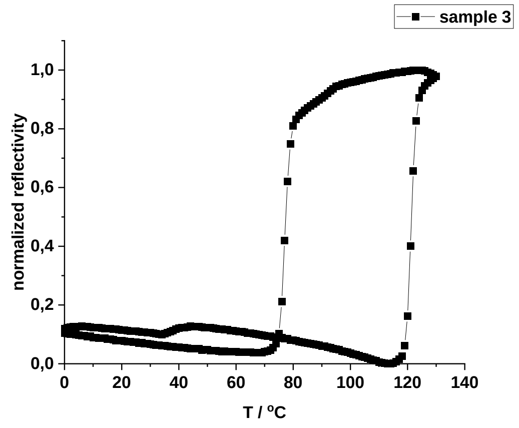
sample 3
normalized reflectivity
T / °C

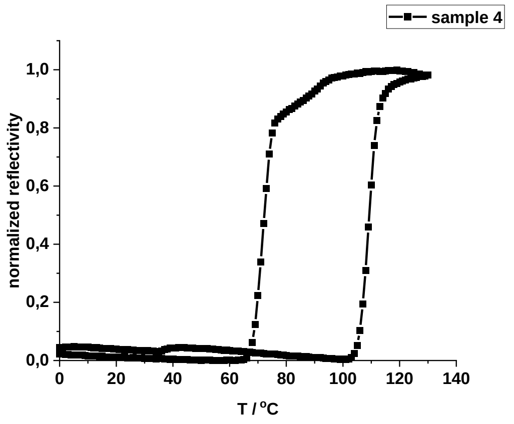
sample 4
normalized reflectivity
T / °C

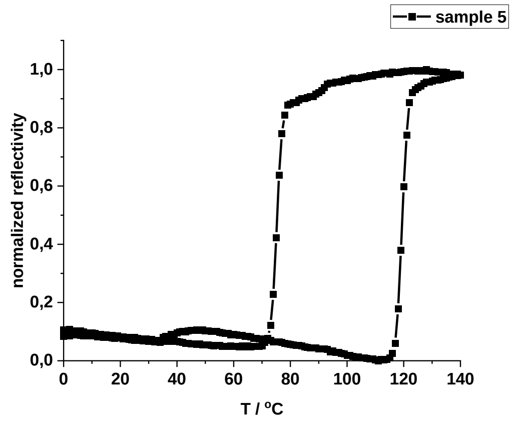
sample 5
normalized reflectivity
T / °C

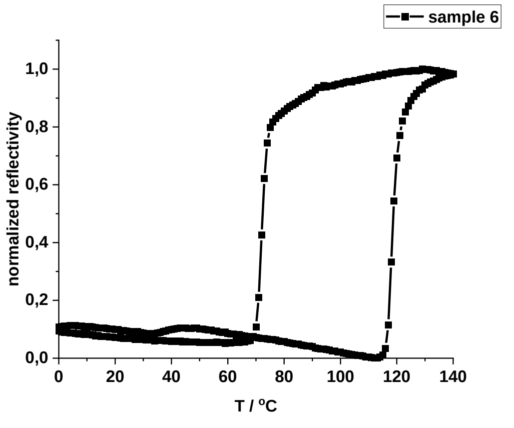
sample 6
normalized reflectivity
T / °C

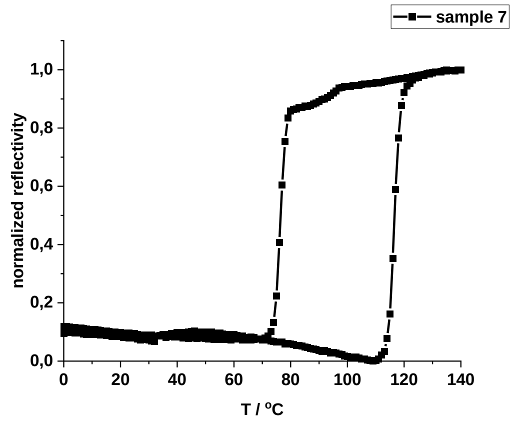
sample 7
normalized reflectivity
1,0
0,8
0,6
0,4
0,2
0,0
0
20
40
60
80
100
120
140
T / °C

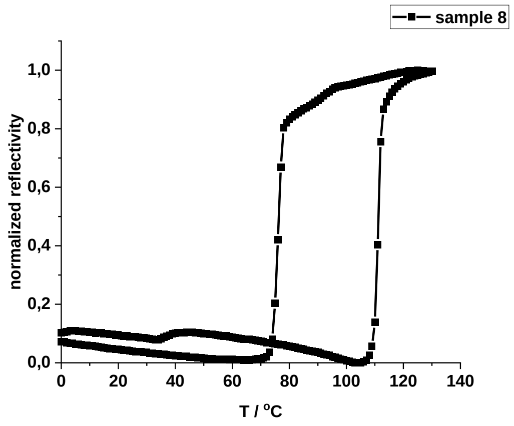
sample 8
normalized reflectivity
1,0
0,8
0,6
0,4
0,2
0,0
0
20
40
60
80
100
120
140
T / °C

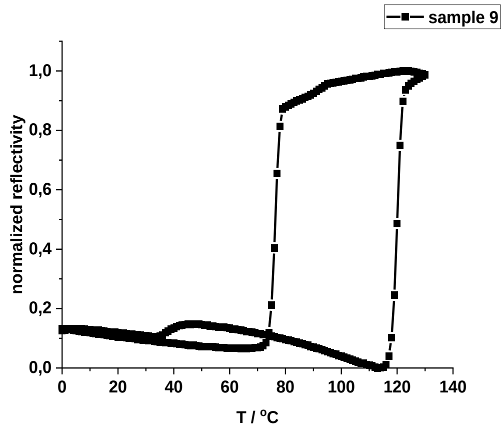
sample 9
normalized reflectivity
1,0
0,8
0,6
0,4
0,2
0,0
0
20
40
60
80
100
120
140
T / °C

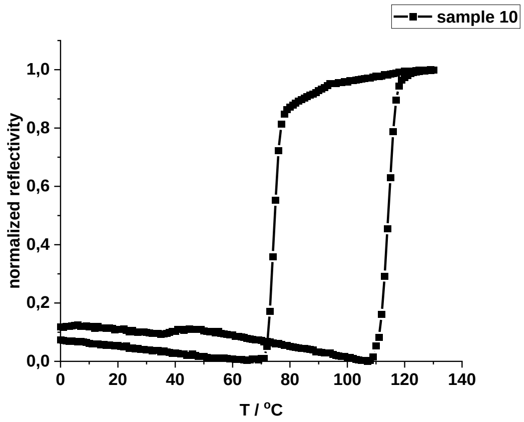
sample 10
normalized reflectivity
1,0
0,8
0,6
0,4
0,2
0,0
0
20
40
60
80
100
120
140
T / °C

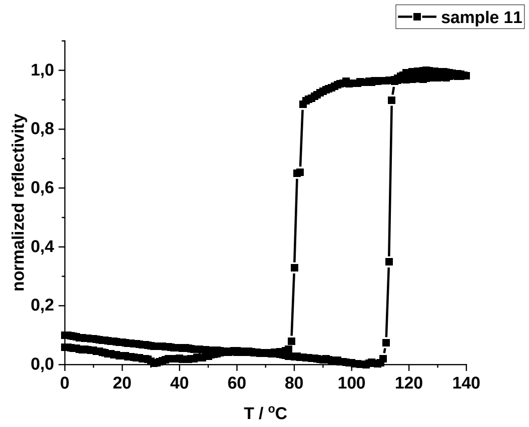
sample 11
normalized reflectivity
1,0
0,8
0,6
0,4
0,2
0,0
0
20
40
60
80
100
120
140
T / °C

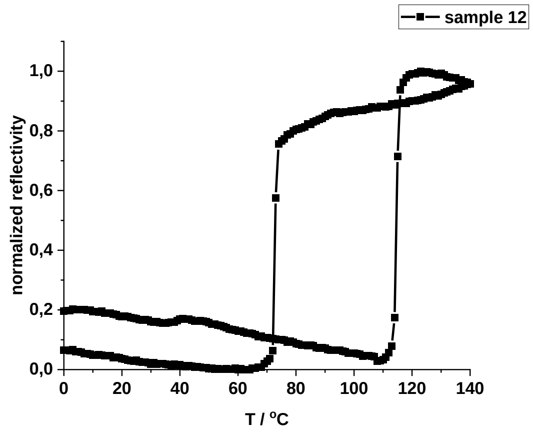
sample 12
normalized reflectivity
1,0
0,8
0,6
0,4
0,2
0,0
0
20
40
60
80
100
120
140
T / °C

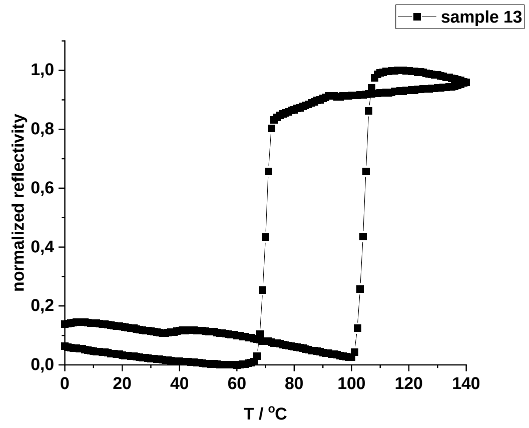
sample 13
normalized reflectivity
1,0
0,8
0,6
0,4
0,2
0,0
0
20
40
60
80
100
120
140
T / °C

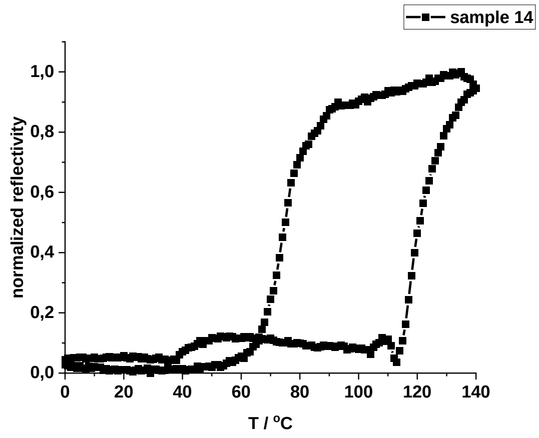
sample 14
normalized reflectivity
1,0
0,8
0,6
0,4
0,2
0,0
0
20
40
60
80
100
120
140
T / °C

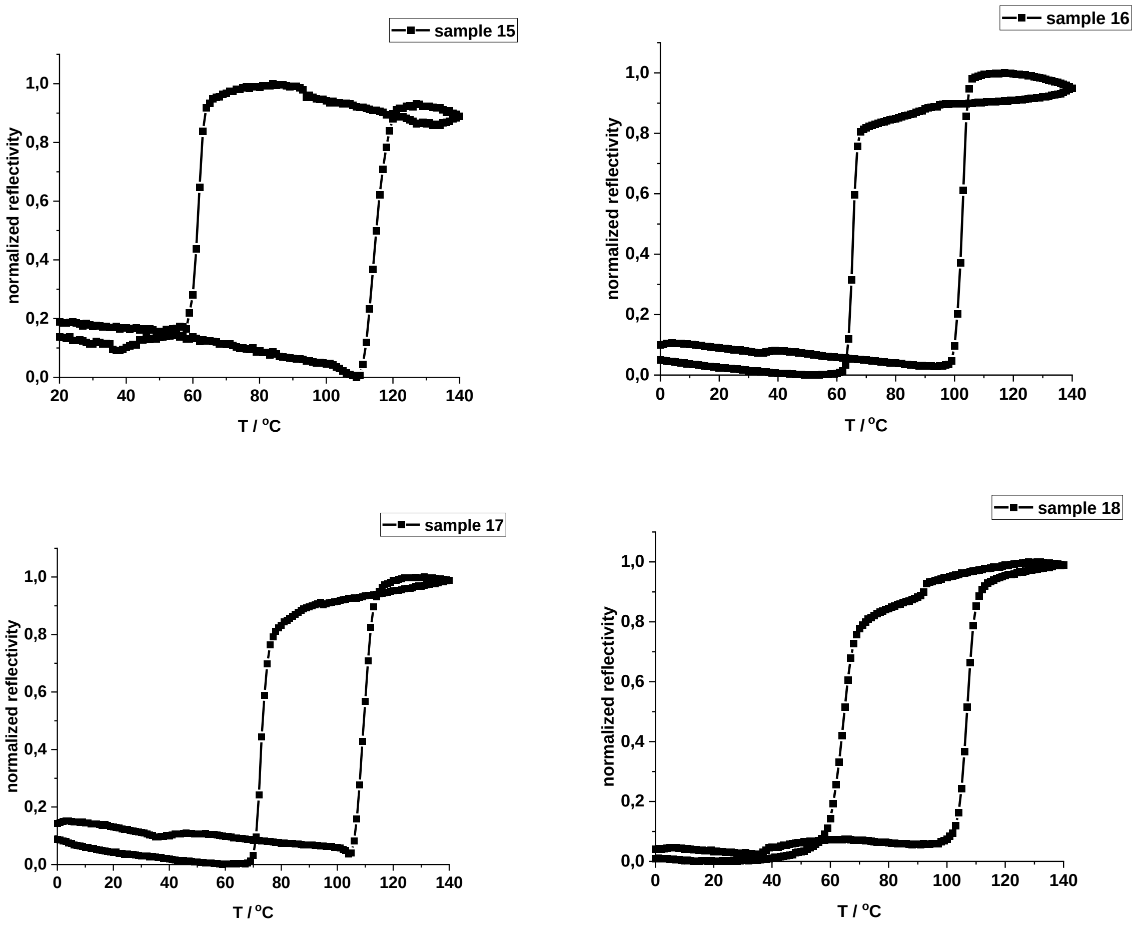


**Figure S4**: Optical reflectivity measurements for samples 1-18
